# Active Video Games for Rehabilitation in Respiratory Conditions: Systematic Review and Meta-Analysis

**DOI:** 10.2196/10116

**Published:** 2019-02-25

**Authors:** Joshua Simmich, Anthony J Deacon, Trevor G Russell

**Affiliations:** 1 Centre for Research Excellence in Telehealth University of Queensland Brisbane Australia; 2 School of Health and Rehabilitation Sciences University of Queensland Brisbane Australia; 3 School of Electrical Engineering and Computer Science Queensland University of Technology Brisbane Australia

**Keywords:** video games, exercise, physical activity, COPD, cystic fibrosis, asthma

## Abstract

**Background:**

Exercise and physical activity are key components of treatment for chronic respiratory diseases. However, the level of physical activity and adherence to exercise programs are low in people with these diseases. Active video games (AVGs) may provide a more engaging alternative to traditional forms of exercise.

**Objective:**

This review examines the effectiveness of game-based interventions on physiological outcome measures, as well as adherence and enjoyment in subjects with chronic respiratory diseases.

**Methods:**

A systematic search of the literature was conducted, with full texts and abstracts included where they involved an AVG intervention for participants diagnosed with respiratory conditions. A narrative synthesis of included studies was performed. Additionally, meta-analysis comparing AVGs with traditional exercise was undertaken for 4 outcome measures: mean heart rate (HR) during exercise, peripheral blood oxygen saturation (S_p_O_2_) during exercise, dyspnea induced by the exercise, and enjoyment of the exercise.

**Results:**

A total of 13 full-text papers corresponding to 12 studies were included in the review. Interventions predominantly used games released for the Nintendo Wii (8 studies) and Microsoft Xbox Kinect (3 studies). There were 5 studies that examined the acute effects of a single session of AVGs and 7 studies that examined the long-term effects after multiple sessions of AVGs. Trials conducted over more than 1 session varied in duration between 3 and 12 weeks. In these, AVG interventions were associated with either similar or slightly greater improvements in outcomes such as exercise capacity when compared with a traditional exercise control, and they also generally demonstrated improvements over baseline or nonintervention comparators. There were a few studies of unsupervised AVG interventions, but the reported adherence was high and maintained throughout the intervention period. Additionally, AVGs were generally reported to be well liked and considered feasible by participants.

For outcome measures measured during a single exercise session, there was no significant difference between an AVG and traditional exercise for HR (mean difference 1.44 beats per minute, 95% CI –14.31 to 17.18), S_p_O_2_ (mean difference 1.12 percentage points, 95% CI –1.91 to 4.16), and dyspnea (mean difference 0.43 Borg units, 95% CI –0.79 to 1.66), but AVGs were significantly more enjoyable than traditional exercise (Hedges *g* standardized mean difference 1.36, 95% CI 0.04-2.68).

**Conclusions:**

This review provides evidence that AVG interventions, undertaken for several weeks, can provide similar or greater improvements in exercise capacity and other outcomes as traditional exercise. Within a single session of cardiovascular exercise, an AVG can evoke similar physiological responses as traditional exercise modalities but is more enjoyable to subjects with chronic respiratory diseases. However, there is very limited evidence for adherence and effectiveness in long-term unsupervised trials, which should be the focus of future research.

## Introduction

### Background

Chronic respiratory diseases such as asthma [1], chronic obstructive pulmonary disease (COPD) [2], and cystic fibrosis (CF) [3] represent a significant disease burden globally, accounting for approximately 7% of all global deaths [4]. Recent systematic reviews have shown physical activity and exercise to be beneficial for pulmonary function and health-related quality of life in both asthma [5] and CF [6]. Physical activity is also negatively associated with mortality and exacerbations in COPD [7]. Exercise is a key component of pulmonary rehabilitation for COPD, which a recent systematic review has shown to improve quality of life and exercise capacity in subjects [8]. Exercise-based pulmonary rehabilitation can also be an effective intervention for other respiratory conditions [9,10]. Exercise is prescribed in clinical guidelines for both CF [11] (grade B recommendation) and COPD [12] (strong recommendation).

Physical activity is defined as any bodily movement resulting in energy expenditure, and exercise is a subset of physical activity that is planned, structured, repetitive, and aimed at improving or maintaining physical fitness [13]. Despite the clear evidence for physical activity and exercise in the management of respiratory conditions, adherence in these populations is generally low. Objective measurements indicate that almost half of adults with CF do not achieve 30 minutes of physical activity per day [14]. Similarly, in COPD, pulmonary rehabilitation has attendance and completion rates as low as 70% and 40%, respectively [15,16], and a recent meta-analysis has shown people with COPD are generally severely physically inactive [17]. Low motivation has been identified as a key internal barrier to physical activity in asthma [18], COPD [19], and CF [20], and enjoyment of physical activity has been shown to be a key motivator of exercise in these populations [20-22].

Video games have attracted attention as a novel way of delivering health care interventions with the potential to motivate change in health behaviors [23]. Video games can be designed to incorporate a physical activity or exercise component, and these are known as active video games (AVGs) or exergames [24]. The use of AVGs has been demonstrated in many age groups and clinical conditions as an enjoyable form of rehabilitation [25,26] and can also be designed to encompass other treatments such as airway clearance or spirometry [27-29]. Games used in rehabilitation may either be designed specifically for this purpose or be commercial games designed for recreational exercise that have been adapted by clinicians and researchers for use in rehabilitation.

Reviews of the use of games for purposes such as improving balance in the elderly, rehabilitation of motor function in stroke, or rehabilitation of upper limb functioning in cerebral palsy, have found small effects in favor of games over traditional therapies [25,26]. Several advantages of video games over traditional rehabilitation have been posited, such as objective measurement of performance and progress (eg, time spent playing the game, number of repetitions performed), low cost, and improved motivation and adherence [30]. The results of individual studies have reported that a single session of an AVG was considered more enjoyable than traditional exercise in subjects with CF [31,32] and COPD [33], in keeping with a systematic review that concluded that AVGs are considered enjoyable by many different populations of subjects undergoing rehabilitation [26]. Given the significant positive correlation between emotional judgment (such as enjoyment) of physical activity and frequency of physical activity in healthy adults [34,35], it is likely that interventions that improve enjoyment of exercise will improve adherence to exercise. In healthy populations, studies have found a correlation between enjoyment of an AVG and exercise adherence [36] or energy expenditure during play [37]. However, the relationship among games, enjoyment, adherence, and overall effectiveness is poorly understood, especially in chronic respiratory conditions.

To date, only 1 systematic review specifically examining the use of video games in respiratory conditions exists. This review was limited in scope as it only aimed to review the exercise intensity of AVGs for people with CF [38], and therefore it only included 5 studies. Another broad review of video games in rehabilitation did not include respiratory conditions and instead focused on aging and pathologies such as stroke and Parkinson disease for which there was more evidence [39]. Other broad reviews have focused only on pathologies for which there are randomized controlled trials looking at changes in rehabilitation outcomes over time [25] or only on games aimed at improving motor function rather than cardiorespiratory function [26]. Reviews that have included respiratory conditions have been limited to scoping reviews rather than systematic reviews [40,41].

### Objectives

By comparing multiple diseases, this review provides a broader picture of the use of video games for rehabilitation in respiratory conditions than previous reviews. Moreover, it examines evidence more specific to the posited advantages of video games in rehabilitation by investigating not only clinical effectiveness of video games but also their economic feasibility and reported measures of adherence and enjoyment. Additionally, it should provide clinicians and researchers with a unified view of the literature on this newly emerging field of game-based pulmonary rehabilitation. The primary aim of this review was to evaluate the effectiveness of AVGs for 3 categories of outcomes: (1) clinical outcomes, (2) economic feasibility, and (3) patient enjoyment and adherence. The secondary aim was to compare AVGs with traditional exercise for the aforementioned outcome measures. This review will examine the hypotheses that (1) AVGs improve clinical outcomes and are economically feasible and enjoyable and (2) that AVGs produce equivalent clinical outcomes as traditional exercise programs but are more cost-effective, more enjoyable, and have higher adherence.

## Methods

### Search Strategy

Studies were identified by searching the following electronic databases: PubMed, Scopus, Web of Science, EMBASE, Cumulative Index to Nursing and Allied Health Literature (CINAHL), and Institute of Electrical and Electronics Engineers (IEEE) Xplore. Medical subject headings terms and equivalent subject headings were used for PubMed, EMBASE, CINAHL, and IEEE Xplore. Search terms for AVGs (eg, “video games,” “computer games,” “digital games,” and “gamification”) were combined with terms for respiratory diseases (eg, “lung,” “pulmonary,” “respiratory,” “airway,” “cystic fibrosis,” and “COPD”) derived from key terms for respiratory diseases listed in the Cochrane Airways Group Search Strategies document [42]. For IEEE Xplore, broader search terms were found to be suitable. There were no date or language restrictions placed on the search, though all search terms were in English. Full details of the final search strategies for each database are available in Multimedia Appendix 1.

The search was completed on October 30, 2017, and each database was searched from inception to the date of search. Search results were imported into Zotero (Version 4.0, George Mason University) and EndNote (Version 18.0, Clarivate Analytics) to automatically remove duplicate citations, and the results were further searched by hand to remove remaining duplicates. The combined list of citations was then imported into Covidence, a Web-based review manager (Veritas Health Innovation) for screening of titles, abstracts, and full texts. Hand-searches of reference lists and citation searches were also conducted for literature citing included studies.

### Study Selection

The inclusion and exclusion criteria are outlined in Textboxes 1 and 2. Two reviewers (JS and AD) independently screened the titles and abstracts of identified papers according to inclusion and exclusion criteria, and potentially relevant papers were identified for full-text screening. Full-text papers were retrieved and screened independently by these 2 reviewers, and reasons were given for all excluded citations. In both stages of screening, disagreements were resolved by consensus or in consultation with a third reviewer (TR) if disagreement persisted.

### Data Extraction and Quality Assessment

One reviewer (JS) extracted data and assessed the quality of each included study. The extracted data and the results of quality assessment were then independently checked by a second reviewer (AD) for accuracy and completeness. Quality was assessed using the *Checklist for Measuring Quality* assessment tool by Downs and Black [43]. Where multiple papers reported data from the same study and cohort, they were considered as 1 study for the purposes of data extraction and quality assessment.

### Data Synthesis

Only studies that compared AVGs with a control exercise rather than an exercise test were included in the meta-analysis (quantitative synthesis) and only for outcome measures reported at similar time points in at least 3 different studies. A narrative synthesis was undertaken for all other studies.

A random effects model was used to assess all outcome measures. The method of Hartung-Knapp-Sidik-Jonkman (HKSJ) [44,45] was employed. This method is recommended over the more common DerSimonian and Laird (DSL) method [46,47] for meta-analyses where only few studies are available (the DSL method was also calculated for comparison). Pooled effect sizes were reported as weighted mean differences among treatments in the original units when possible, with a 95% CI. If studies used different scales, effect sizes were expressed as standardized mean differences using Hedges *g* [48], also with a 95% CI. Statistical heterogeneity was estimated using the point estimate and 95% CI of the *I*^2^ statistic.

**Inclusion criteria.**

- Is an experimental or quasi-experimental study of an intervention including quantitative data. This includes studies of the following designs: randomized controlled trials, quasi-randomized trials, interrupted time series, controlled before-and-after studies, uncontrolled before-and-after studies
- Participants are predominantly people diagnosed with respiratory conditions. Respiratory conditions include any disease under the National Library of Medicine Medical Subject Headings term “Respiratory Tract Diseases” (C08), including lung cancer (C08.381.540), cystic fibrosis (C08.381.187), asthma (C08.127.108), and chronic obstructive pulmonary disease (C08.381.495.389).
- Includes an intervention involving any form of video game, console game, virtual reality game, or gamified mobile app or computer program that requires players to perform a physical action that forms part of their rehabilitation, such as perform cardiovascular exercise, use a spirometer, perform airway-clearance techniques

**Exclusion criteria.**

- Study is not experimental or does not include quantitative data. Therefore, publications of the following designs are excluded from descriptive and observational studies (case series, case-control, cohort studies, and qualitative research designs), secondary research studies (reviews and meta-analyses), and protocol papers
- Includes only interventions that do not require participants to physically perform rehabilitative actions in order to progress in the game, such as educational games that merely encourage or teach the importance of consistent medication use or physical activity
- Is not a full-text paper published in a peer-reviewed journal

Additionally, 90% CI were calculated as above for those outcome measures where included studies attempted to demonstrate equivalence among interventions (heart rate, dyspnea, and peripheral blood oxygen saturation [S_p_O_2_]). To demonstrate equivalence with α=.05, the 90% CI must lie between the predetermined equivalence margins [49]. For heart rate (HR) evoked during exercise, we used a difference of 10 beats per minute (bpm) (ie, –10 to +10 bpm) for our equivalence margins as there is no agreed-upon minimum important difference, and 2 previous studies nominated a difference of 10 bpm as important when performing their initial sample size calculations [31,32]. For dyspnea, equivalence margins of 1 unit (ie, –1 to +1) were used, as it has been suggested that the minimum clinically important difference for the modified 11-point (0-10) Borg scale is 1 unit [50]. For S_p_O_2_, a drop of 4% or more has been used as a definition for exertional oxygen desaturation in CF [51,52] and COPD [53]; therefore, the width of our equivalence margins was set at 4% (ie, –2% to 2%).

Data from crossover trials were treated as paired data in the meta-analysis where raw mean differences could be calculated. An estimate of the within-subject SD was estimated where possible from the reported CIs of the difference among treatments or *P* values from a paired *t* test of the difference among treatments. From the within-subject SD, a within-subject correlation was calculated using methods outlined by Elbourne et al [54]. Where a within-subject correlation could not be calculated, the correlation from another study of similar design was used as an estimate. A sensitivity analysis, where this correlation was altered to either 0 (uncorrelated) or 1 (perfectly correlated), was conducted to test the influence this assumption had on the pooled estimate. Where results had to be reported as standardized mean differences, because of comparison across different scales, data from crossover trials were treated as if it came from independent groups.

Data reported only as median and interquartile range were converted to mean and SD using formulas from Wan et al [55]. Ordinal data, such as that from Likert or Likert-type items, were treated as interval data for the purposes of this meta-analysis. Authors of the studies included in the meta-analysis were contacted for additional information, and, when provided, these additional data were used in place of the summary data reported in the publications.

Data conversions and statistical analysis were performed using R software (Version 3.4.0, R Foundation for Statistical Computing), with meta-analysis using the *metafor* package (Version 1.9-9, Viechtbauer) [56].

Code used to generate the analysis and sections of the paper was written as a dynamic manuscript, using the R package *rmarkdown*, version 1.3 (RStudio Inc) [57], and it is available in Multimedia Appendix 2 for reproducing all data conversions and statistical analysis. Data used in this manuscript are presented in Multimedia Appendices 3 and 4.

## Results

### Study Selection

**Figure 1:**
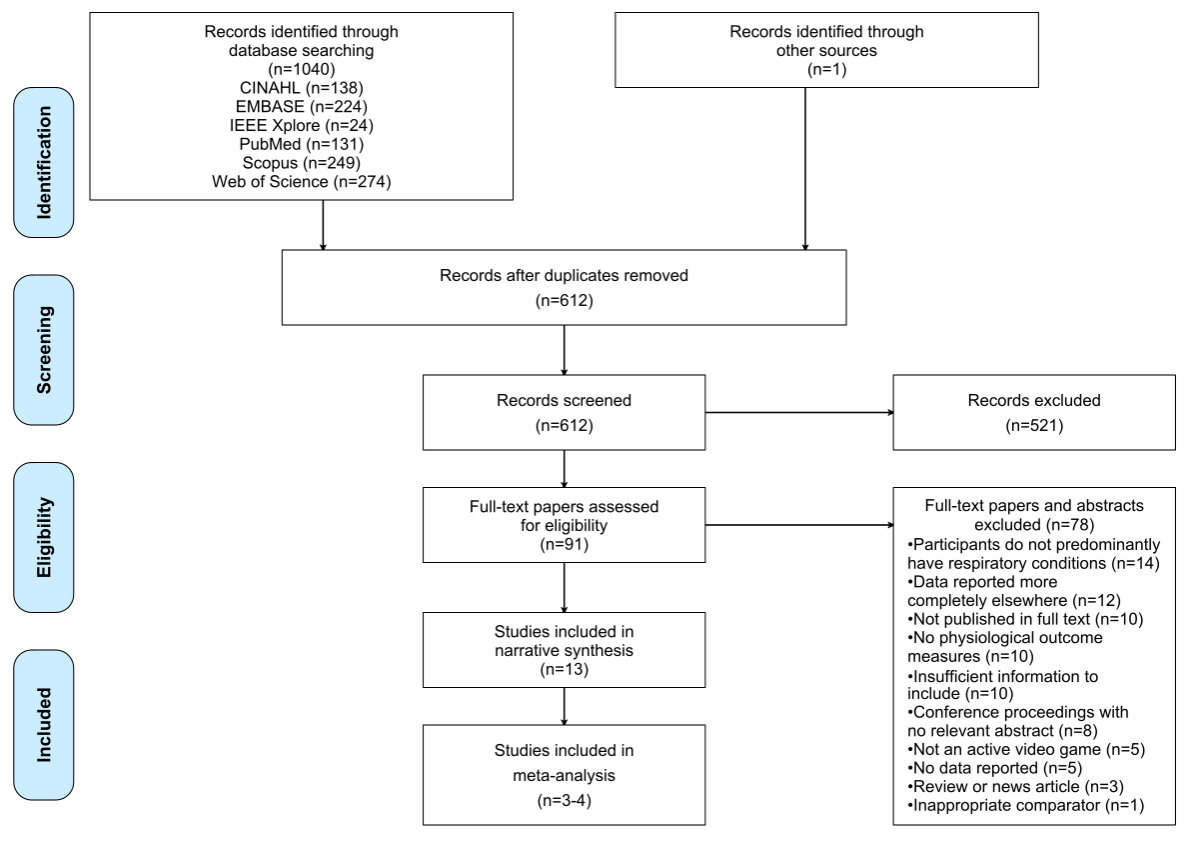
Preferred Reporting Items for Systematic Reviews and Meta-Analyses flow diagram of the search process. CINAHL: Cumulative Index to Nursing and Allied Health Literature; IEEE: Institute of Electrical and Electronics Engineers.

Figure 1 shows the Preferred Reporting Items for Systematic Reviews and Meta-Analyses (PRISMA) flow diagram [58], which illustrates the screening and selection process. The initial literature search gave a total of 1040 results, with 612 unique records remaining after duplicates were removed—PRISMA flow diagram [58] shown in Figure 1. After screening titles and abstracts, 91 results that met the inclusion criteria remained for full-text assessment. A total of 13 papers passed both the inclusion and exclusion criteria after full-text assessment and were included in the narrative synthesis, which corresponded to 12 studies. The interventions and outcomes studied were heterogeneous, with the meta-analysis including only 4 studies for HR, 3 studies for mean S_p_O_2_, 3 studies for reported dyspnea, and 3 studies for enjoyment of the exercise interventions. Manual screening of titles listed in the reference lists of included full-text papers did not yield any additional papers. One potential paper was found by citation tracking but did not pass full-text screening.

### Study Characteristics

There were a total of 292 participants in the 12 full-text papers. Sample sizes ranged from a small observational study of 7 to larger studies with 40 participants (parallel-groups design), 30 participants (crossover-trial), and 32 participants (observational study). In total, 6 studies included participants with CF [27,31,32,59-61], 3 studies included participants with COPD [33,62,63], and 3 with other or mixed respiratory conditions [64-66]. Ages were varied because of the respiratory conditions of interest: studies of asthma included only children, studies of CF included children or young adults, and studies of lung cancer and COPD included only older adults. The randomized experimental studies often used crossover designs [27,31-33,59], with only 3 studies [60,64,66] employing 2 parallel-groups design. Nonrandomized and observational studies represented the remaining 4 studies [61-63,65]. Additional details about each included study, including the specific games played on each device, can be found in Multimedia Appendix 5.

Interventions were predominantly commercially available off-the-shelf AVGs, namely games released for the Nintendo Wii and Microsoft Xbox Kinect. The Wii was used in 8 out of 12 studies [31,33,59,60,62,63,65,66], whereas the Kinect was used in 3 out of 12 studies [32,61,64]. Only 1 study reported the testing of custom-designed hardware and games for respiratory conditions [27].

A total of 4 out of 12 included studies reported randomized studies examining the long-term effects of AVGs versus traditional training in respiratory conditions [27,60,64,66]. One study examined the effect of AVGs versus treadmill training in asthma, with training sessions twice a week over 8 weeks [64]. Another examined the effect of AVGs in combination with usual pulmonary rehabilitation compared with usual pulmonary rehabilitation alone, with 3 weeks of pulmonary rehabilitation in both groups but 1 week of AVGs in the final week of the experimental group [66]. A third compared a custom spirometer game with a control device over a 2- to 3-week period, with participants prompted but not required to use the device [27]. There were also 3 studies [62,63,65] that looked at longer-term effects of AVGs but were not randomized and controlled experiments.

There were 5 studies [31-33,59,61] that examined acute effects of a single session of an AVG. For most of these, the AVG was compared with traditional exercises [31-33,64], though some compared AVGs with maximal [61] or submaximal exercise tests [59].

### Quality Assessment

Multimedia Appendix 6 describes the quality of the included studies as assessed on the Downs and Black checklist.

Due to the nature of the intervention, no study blinded participants to treatment and only 3 blinded those assessing all outcome measures [27,60,64]. Additionally, none attempted to demonstrate that their participants were representative of the population or that their participants were recruited randomly. Only 4 studies reported concealing patient allocation until the experiment was complete [31,32,60,64]. Only 6 studies reported adequate statistical power [31,32,60,61,64,66], though 1 study did perform a power calculation but was underpowered because of dropouts [62].

### Meta-Analysis

A meta-analysis comparing AVGs with traditional exercise was only able to be conducted for 4 outcome measures: mean HR during exercise, S_p_O_2_ during exercise, dyspnea induced by the exercise, and enjoyment of the exercise.

#### Mean Heart Rate

**Figure 2:**
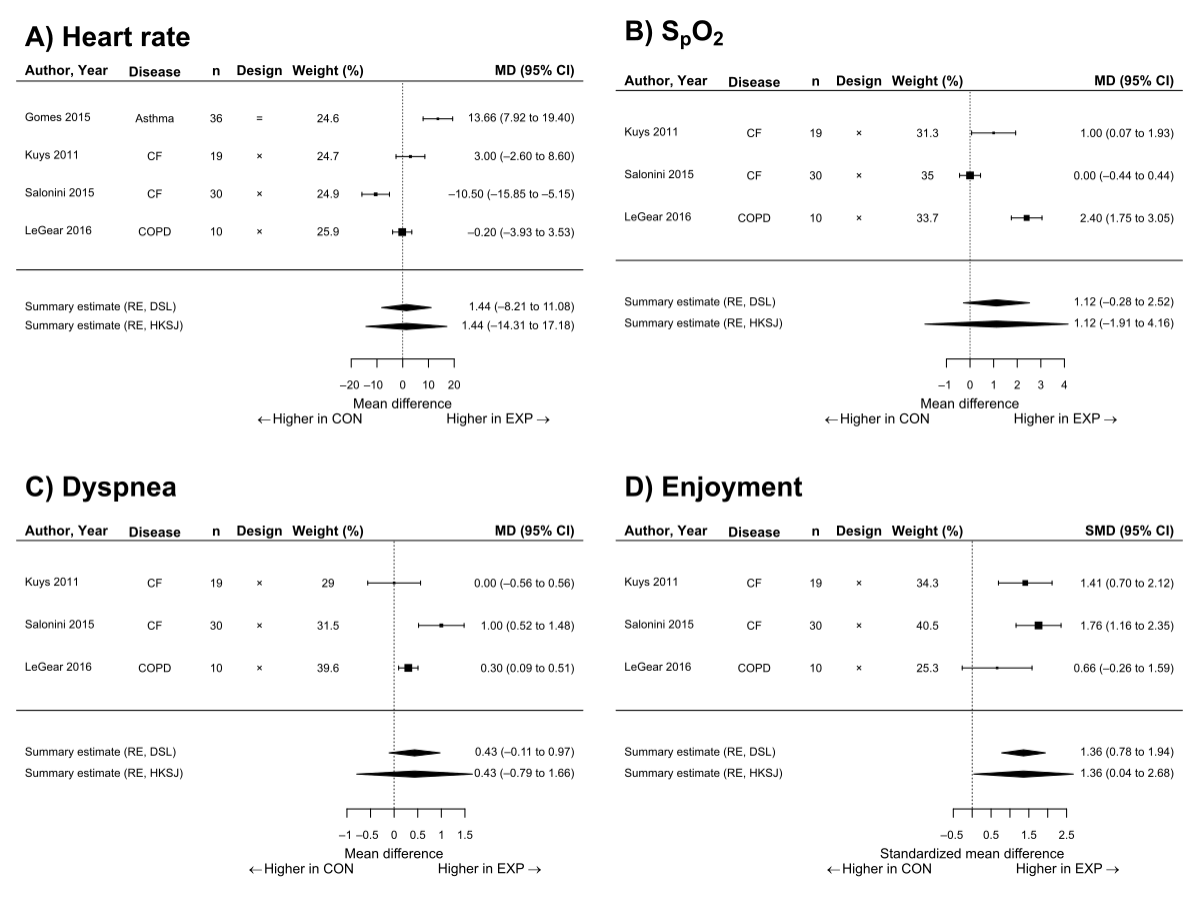
Forest plots showing comparisons among multiple outcomes reported during exercise in the experimental active video game conditions or the traditional exercise control conditions. MD: mean difference; CF: cystic fibrosis; COPD: chronic obstructive pulmonary disease; RE: random effects; DSL: DerSimonian-Laird; HKSJ: Hartung-Knapp-Sidik-Jonkman; EXP: experimental; CON: control; S_p_O_2_: peripheral blood oxygen saturation.

The mean HR of AVGs and traditional exercise was assessed in 4 studies with a total of 95 participants. As shown in Figure 2, the mean difference between mean HR during AVGs and mean HR during traditional exercise was 1.44 (95% CI –14.31 to 17.18) bpm, indicating there was no significant difference between the 2 interventions. Statistical heterogeneity by *I*^2^ was found to be 93% (95% CI 79%-99%).

The lower and upper bounds of the 90% CI for the difference in HR between interventions are –10.21 bpm and 13.08 bpm, respectively, which are not within the equivalence margins of –10 bpm and 10 bpm. Therefore, these treatments cannot be considered equivalent at α=.05 level.

#### Mean Peripheral Blood Oxygen Saturation

The mean S_p_O_2_ of AVGs and traditional exercise was assessed in 3 studies with a total of 59 participants. As shown in Figure 2, the mean difference between mean S_p_O_2_ during AVGs and mean S_p_O_2_ during traditional exercise was 1.12 (95% CI –1.91 to 4.16) raw percentage points, indicating there was no significant difference between the 2 interventions. Statistical heterogeneity by *I*^2^ was found to be 93% (95% CI 74%-100%).

The lower and upper bounds of the 90% CI for the difference in mean S_p_O_2_ between interventions are –0.94% and 3.18%, respectively, which are not within the equivalence margins of –2% and 2%. Therefore, these treatments cannot be considered equivalent at α=.05 level.

#### Dyspnea

The mean dyspnea of AVGs and traditional exercise was assessed in 3 studies with a total of 59 participants. As shown in Figure 2, the mean difference between mean reported dyspnea during AVGs and during traditional exercise was 0.43 (95% CI –0.79 to 1.66) points on the modified 11-point (0-10) Borg scale, indicating there was no significant difference between the 2 interventions. Statistical heterogeneity by *I*^2^ was found to be 81% (95% CI 18%-100%).

The lower and upper bounds of the 90% CI for the difference in reported dyspnea between interventions are –0.4 and 1.27 points on the modified 11-point (0-10) Borg scale, respectively, which are not within the equivalence margins of –1 and +1 points. Therefore, these treatments cannot be considered equivalent at α=.05 level.

#### Enjoyment

Measures of enjoyment of AVGs and traditional exercise were reported in 3 studies with a total of 59 participants. As shown in Figure 2, the standardized mean difference between mean reported enjoyment during AVGs and during traditional exercise was 1.36 (95% CI 0.04-2.68), using Hedges *g*, indicating there was a significant difference between the 2 interventions. Statistical heterogeneity by *I*^2^ was found to be 47% (95% CI 0%-99%).

In the absence of other information, Cohen proposed that standardized effect sizes (such as Cohen *d* or Hedges *g*) of greater than or equal to 0.8 be considered as large effects [67]. The effect of enjoyment of AVGs observed in this meta-analysis would therefore be considered a large effect.

### Sensitivity Analysis

A sensitivity analysis was conducted by altering the correlation coefficients of crossover studies where the correlation could not be calculated, which had been assumed to be equal to the correlation calculated in another similar study. This assumption had negligible influence on the outcome measures of HR and enjoyment and little influence on the results of dyspnea (see Multimedia Appendix 7 for full analysis).

### Narrative Synthesis

#### Active Video Games Versus Exercise Test Protocols

Results from 2 studies examining the physiological responses to a single session of active video gaming compared with maximal exercise tests (eg, cardiopulmonary exercise testing and incremental shuttle walk test) suggest that video games usually provide a less intense exercise stimulus than a maximal exercise text. Holmes et al [61] compared the intensity of exercise elicited from the Microsoft Xbox Kinect with the maximal exercise capacity of participants with CF and found the average HR achieved during the 10-min video game session was 86% (95% CI 81-92) of the peak HR achieved during a cardiopulmonary exercise test (using a modified Godfrey Cycle Ergometer Protocol). From this, they conclude the game represented an exercise intensity of 6.1 (SD 1.8) metabolic equivalents (METS), just above the 6METS threshold for vigorous intensity activity in healthy adults [68]. They also found the game produced lower reported dyspnea, lower rating of perceived exertion, and caused less oxygen desaturation than the maximal exercise test.

Another study compared AVGs with a submaximal exercise test, such as a 6-min walk test (6MWT). Del Corral et al (2014) [59] performed a randomized cross-over observational trial to compare the physiological responses with 5 min of 3 different AVGs using the Nintendo Wii console and a 6MWT (repeated twice). They found the 2 games that were focused on aerobic exercise (*EA Sports Active* and *Family Trainer Extreme Challenge*) both elicited a higher volume of oxygen consumption (VO_2_) than the 6MWT. However, the game that included balance exercises in addition to aerobic exercises (*Wii Fit* *Plus*) elicited a lower VO_2_ during the final 3 min of exercise than the 6MWT. HR was also lower in the *Wii Fit* *Plus* game than the 6MWT but did not differ among the 6MWT and the other 2 games, and there was no statistically significant difference in dyspnea among any intervention.

#### Active Video Games Versus Rest Only

One study compared physiological responses with AVGs to rest without comparison with a control exercise. The average response to several Nintendo Wii gaming sessions in subjects with COPD was presented by Wardini et al, showing that games significantly increased HR (by a mean of 13.8 bpm) and dyspnea and decreased S_p_O_2_ compared with rest.

#### Long-Term Physiological Effects of Active Video Games

In total, 7 studies included more than 1 session of an AVG intervention. When compared with those seen in a control intervention, game interventions were associated with either similar or slightly greater improvements in measured outcomes. For example, in a randomized controlled trial with parallel groups, Gomes et al [64] reported both Microsoft Xbox Kinect and treadmill training for 8 weeks improved aerobic capacity and asthma control, but only the game intervention showed a statistically significant improvement in exhaled fraction of nitric oxide. Similarly, Bingham et al [27] reported forced expiratory volume in 1 second was better maintained during the game intervention than the control intervention. Mazzoleni et al [66] reported both 6MWT distance and dyspnea improved more in the participants performing pulmonary rehabilitation with an adjunct AVG than pulmonary rehabilitation alone.

AVG interventions generally demonstrated improvements over baseline or nonintervention comparators. In a randomized 6-week trial, del Corral et al [60] reported the Nintendo Wii group, but not the control group receiving no intervention, improved both modified shuttle walk test and 6MWT distances, as well as several measures of strength. Similarly, in a single-arm study, Albores et al (2013) [62] reported a statistically significant improvement in endurance shuttle walk test, number of sit-to-stands in 30 seconds and number of arm lifts in 30 seconds after 12 weeks of unsupervised Nintendo Wii training in subjects with COPD. In another single-arm study, Hoffman et al (2013) [65] reported an increase in functional performance, as measured by the number of steps per day, during the game intervention period. No studies reported AVGs were inferior to control or were associated with a negative change to an outcome measure relative to baseline. Wardini et al [63] performed a 3- to 4-week intervention but did not report changes in outcome measures over time.

#### Adherence and Patient Preference

A total of 5 studies examined adherence to unsupervised AVG treatments and generally reported adherence was higher than 70% and maintained throughout the intervention period [27,60,62,63,65]. The controlled trial by del Corral [60] reported the adherence to the home intervention was 95% during the 6-week trial, but adherence dropped in the 12-month follow-up with 65% no longer using the AVG at all. A single-arm study by Hoffman et al (2013) [65] reported a rate of adherence of 96.6% (SD 3.4%) to a Nintendo Wii intervention in subjects who had undergone surgery for suspected lung cancer. Moreover, the mean time spent performing a walking exercise with the Wii increased during the first 6 weeks’ postsurgical recovery period and was maintained for the following 10 weeks [69]. In their uncontrolled (single-arm) study, Albores et al (2013) [62] instructed subjects with COPD to use a Nintendo Wii for exercise for at least 30 minutes on most days of the week. Subjects self-reported exercising at the prescribed frequency and duration throughout the 12-week intervention, with a frequency of 5.7 (SD 1.0) days per week and a weekly total of 3.0 (SD 0.9) hours. Bingham et al [27] did not prescribe the use of their intervention; therefore, adherence could not be calculated. Nonetheless, their game intervention was used for significantly more minutes per day than the control (4.8 [SD 4.4] vs 1.6 [SD 1.8]), with no difference in the number of days each intervention was used. In contrast, in a study by Wardini et al [63], the attendance rate to the game intervention (64% [SD 35%]) was lower than the attendance rate for the pulmonary rehabilitation program (88% [SD 13%]), though the game was delivered as an adjunct rather than an alternative program and no statistical comparison was performed.

Measures of patient preferences, likeability, or perceived feasibility were measured in several studies (in addition to enjoyment, which was summarized in the meta-analysis), showing that games were generally considered likeable and feasible interventions [31,33,63,66]. For example, Kuys et al [31] reported that both the Nintendo Wii intervention and the treadmill or bicycle control exercise were rated highly effective and feasible without significant differences between the 2 interventions. Similarly, LeGear et al [33] reported similar responses between game exercise and treadmill exercise for perceived safety and whether the participants could see themselves performing that exercise at home. Using a 7-item, 7-level Likert-type scale, Mazzoleni et al [66] reported no significant difference between the acceptability of pulmonary rehabilitation alone (43.9 [SD 3.0])) and with active video gaming as an adjunct (42.4 [SD 3.5]). Wardini et al [63] reported the overall enjoyment of the adjunct game intervention to be 8.0 cm (SD 2.6 cm) on a 10-cm visual analog scale. Moreover, participants gave a mean score of 8.0 (SD 2.6 cm when asked if they would recommend the adjunct program to another patient with COPD and a rating of 7.0 cm (SD 3.7cm) when asked if they would consider purchasing a game system of their own.

#### Adverse Events

No studies reported the occurrence of adverse events linked to AVGs. Wardini et al [63] reported 1 patient with COPD and coronary artery disease requiring the use of nitroglycerin and 5 subjects experiencing transient but asymptomatic oxygen desaturation below 85%, but they did not report whether these were a result of the AVG intervention or the control intervention.

### Economic Analysis

No studies performed an economic analysis comparing AVGs with alternative forms of exercise or rehabilitation.

## Discussion

### Summary of Findings

This systematic review aimed to examine the hypotheses that (1) AVGs improve clinical outcomes and are economically feasible and enjoyable and (2) that AVGs produce equivalent clinical outcomes as traditional exercise programs but are more cost-effective, more enjoyable, and have higher adherence. AVGs generally demonstrated improvements in exercise capacity or functional performance over baseline or nonintervention comparators, and they were generally reported to be well liked by participants. There were no differences among AVGs in physiological outcomes when compared with traditional exercises (such as treadmill or stationary cycling) in the laboratory over a single session, but AVGs were enjoyed more by participants. Despite a few long-term trials, the limited evidence suggests AVGs may provide similar or greater effects on clinical outcome measures compared with traditional forms of exercise. None of the included studies provided any evidence for the economic feasibility of the interventions.

The results of this review corroborate the findings of a systematic review into the effects of AVGs on people with CF [38], which found that the HR response to active video gaming was similar to the traditional methods of physical training. One of the studies included in that review was excluded from this review as it did not compare AVGs with traditional exercise or with rest [70]. This study found that the HR target for moderate physical activity (64% of predicted maximum HR) was met during the boxing activity of the Nintendo Wii game *Wii Sports* and exceeded by the free jogging activity in the Nintendo Wii game *Wii Fit* in children with CF.

Similarly, the results of this review are consistent with a meta-analysis of AVGs for healthy adults and children by Peng et al [71], who concluded that the HR, energy expenditure, and oxygen consumption were no different between AVGs and traditional physical activities. In contrast, a more recent meta-analysis on AVGs in healthy children [72] found that AVGs had a large positive mean effect on HR, small positive mean effect size on enjoyment, and a similar rate of perceived exertion compared with traditional exercise in a laboratory setting.

### Physiological Responses to Active Video Games

The results of the meta-analysis indicate that the physiological response to AVGs was not significantly different from traditional exercises (Figure 2). However, statistical equivalence was not demonstrated for any of these physiological outcomes, possibly because of there being so few included studies and the heterogeneity among studies.

The physiological response to AVGs may partly derive from psychological arousal rather than the physical workload of the activity. An increase in the HR and respiratory rate has been observed in healthy adults and children playing inactive video games with traditional controllers [73,74]. Similarly, Sherman et al [75] found that the HR changes were similar whether the game was controlled with a joystick or a motion sensor detecting the movement of the player’s body. However, the HR changes reported in the studies included in this review are greater than those seen in response to inactive video games. Additionally, the pooled effect of AVGs on HR and dyspnea was no different from traditional moderate-intensity exercise; therefore, psychological arousal likely had only a small or negligible contribution to the observed physiological responses.

Furthermore, in each study included in the meta-analysis, the participants were instructed to perform both AVGs and traditional exercises at similar self-perceived intensities and supervised to ensure compliance. For example, in both Kuys et al [31] and Salonini et al [32], the participants in each intervention were instructed to exercise at an intensity corresponding to 3 to 5 on the modified Borg scale. Therefore, it is not known whether these interventions would produce similar workloads in a situation where subjects were unsupervised or allowed to exercise at a self-selected intensity.

Additionally, 1 included study [32] did not use the 11-point (0-10) modified Borg scale used by other included studies but rather a visual analog scale that was also 0 to 10, though the results were nonetheless pooled as if they are equivalent scales. Dyspnea assessed on a visual analog scale correlates strongly with the modified Borg scale [76,77], but the correlation between these 2 assessment scales is not perfect, and it therefore may have been a source of additional variability. Furthermore, Salonini et al [32] assessed dyspnea during exercise, whereas other studies [31,33] assessed dyspnea at the end of the exercise bout. Both of these differences in assessment among studies may have further contributed to the heterogeneity observed in the meta-analysis.

Additionally, the meta-analysis pooled values for mean S_p_O_2_ as this was the most commonly reported measure, but minimum S_p_O_2_ may be more relevant to detecting the occurrence of exertional oxygen desaturation that can occur in CF [51,52] and COPD [53,78]. Only 2 papers reported minimum S_p_O_2_. Kuys et al [31] found that minimum S_p_O_2_ was 2% lower in traditional exercise compared with game-based exercise, but this was not a significant difference (95% CI –2 to 6). LeGear et al [33] reported that minimum S_p_O_2_ was 91.3% during the game versus 88.7% in the treadmill, but they did not perform a statistical comparison between these interventions (nor report sufficient data to enable one to be calculated).

### Other Responses to Active Video Games

The pooled estimate of enjoyment of AVGs was significantly higher than traditional exercises; however, this estimate was based upon a small number of studies with heterogeneous outcome measures. Each included study assessed enjoyment using different measures and none used an experimentally validated measure of enjoyment, which limits comparison among included studies and with the literature as a whole. Established, validated instruments for assessing enjoyment of exercise include the Physical Activity Enjoyment Scale [79] or Feeling Scale [80]. In healthy sedentary adults, an increase of 1 unit on the 11-point bipolar (–5 to +5) Feeling Scale during moderate-intensity treadmill walking is associated with an increase of 15 minutes of weekly physical activity at 6-month follow-up [81], though this result was observational, and causation therefore could not been demonstrated. Nonetheless, it is possible that the 2-unit difference on the 0 to 10 cm visual analog scale in response to AVGs compared with traditional exercise reported by Kuys et al [31], which corresponds with the pooled estimate of the meta-analysis of this study (as shown in Figure 2), could result in at least a similar increase in physical activity in subjects with respiratory diseases.

The included studies assessed enjoyment of exercise by assessing participants after exercise rather than during. Enjoyment assessed after exercise may be biased because of either the time between exercise and giving an enjoyment response or to an emotional response to completion of the task.

Some studies used a continuous mode of exercise as a control (continuous cycling or treadmill walking), whereas participants playing AVGs performed periods of exercise interspersed with rest periods between game levels [32,33]. Interval training has been demonstrated to be more enjoyable than continuous exercise in healthy active populations [82,83], which may explain at least part of the enjoyment effect of AVGs over traditional exercise in the included studies. Additionally, the video game intervention often involved different exercises and a greater variety of exercises compared with the control [31,33]. Thus, it is possible that a control intervention, which also comprised a variety of exercises (eg, dancing, boxing, and running) may have been just as enjoyable as the AVG. In future, studies should compare AVGs with interval training comprising a routine or circuit with an equivalent variety of exercises.

Additionally, several included studies noted that the greater enjoyment found in AVGs may be because participants found them more novel than traditional exercises. Studies of longer duration must be performed to see if enjoyment is maintained in subjects with respiratory diseases using AVGs as part of their rehabilitation and if this corresponds to a greater adherence to exercise. Most studies used supervised exercise training and therefore could not assess the effect of video games on adherence to unsupervised home exercise or self-chosen duration of exercise. The greater enjoyment seen in video games could fail to translate to greater adherence or longer durations of exercise, perhaps because the additional technological requirements of AVGs compared with other modes of exercise (such as jogging) may decrease adherence in a real-world situation.

No adverse events linked to the AVG intervention were reported in any included studies, though the small sample sizes of included studies may have limited their ability to detect rare adverse events.

### Strengths and Limitations

This review includes evidence from multiple respiratory conditions, which provides a broader picture using a greater quantity of evidence than is currently available for any condition individually. Additionally, despite the different methodologies and reported outcome measures, this review transformed and standardized the data as best as possible to enable a meta-analysis to be conducted, using the conservative HKSJ approach [44,45].

The main limitation of this review is the small number and significant heterogeneity of the included studies. This review mostly included relatively small studies that examined multiple different respiratory diseases, within different age groups and using different interventions (eg, types of software and technology). A subgroup analysis was not performed for age groups or disease populations as the evidence was limited to just 1 or 2 studies in each group. This clinical heterogeneity was the reason for performing a narrative synthesis for several outcomes. The statistical heterogeneity was also high; however, because of the low number of studies, even the estimates of statistical heterogeneity were highly uncertain.

An additional limitation is the inclusion of only studies published in full, which ensured data came only from high-quality studies but may have caused the review to be vulnerable to publication bias, which also may have limited previous reviews [26]. Language bias may also have been present as all search terms were in English, although indexed terms were used, which may have minimized potential language bias. Finally, the HKSJ estimation method, though chosen because it provides a more conservative estimate than more common approaches, may overestimate uncertainty with so few included studies [46].

### Conclusions

The results of this systematic review and meta-analysis indicate that in a single session, AVGs, when used for cardiovascular exercise, can evoke similar exercise intensities to those produced by traditional exercise modalities but are considered more enjoyable by subjects with respiratory diseases. However, little evidence exists from unsupervised long-term trials of AVGs used for any rehabilitative purpose in respiratory conditions, though some limited evidence from supervised longer-term trials indicates that AVGs may be equal or slightly superior to traditional exercise training in some outcome measures.

Future research is needed regarding the long-term effects of AVGs, the use of AVGs for other rehabilitative purposes such as strength training or airway clearance techniques, and the economic benefits of utilizing AVGs rather than traditional supervised or unsupervised rehabilitation programs. Additionally, future work could explore the differences between technologies (game consoles) and software (individual games) to enable the creation of more effective gaming interventions for use in rehabilitation. Finally, when assessing the enjoyment of game interventions, validated outcome measures should be used to facilitate comparison to established research on enjoyment of exercise interventions.

## Appendix

Multimedia Appendix 1 Search terms used for each database.

Multimedia Appendix 2 Compressed archive containing source files (in RMarkdown format), plus necessary associated formatting templates and citation files, to generate the submitted manuscript. Requires raw data files (see other appendices).

Multimedia Appendix 3 Raw data for results of database searches and screening process.

Multimedia Appendix 4 Raw data extracted from included studies, for characteristics of included studies and outcome variables for meta-analysis.

Multimedia Appendix 5 Characteristics of included studies.

Multimedia Appendix 6 Study quality assessed using Downs and Black (1998) checklist.

Multimedia Appendix 7 Sensitivity analysis for meta-analysis.

## Abbreviations

AVG: active video game
bpm: beats per minute
CF: cystic fibrosis
CINAHL: Cumulative Index to Nursing and Allied Health Literature
COPD: chronic obstructive pulmonary disease
DSL: DerSimonian-Laird
HKSJ: Hartung-Knapp-Sidik-Jonkman
HR: heart rate
IEEE: Institute of Electrical and Electronics Engineers
METS: metabolic equivalents
6MWT: 6-min walk test
PRISMA: Preferred Reporting Items for Systematic Reviews and Meta-Analyses
S_p_O_2_: peripheral blood oxygen saturation
VO_2_: volume of oxygen consumption
