# Supplementary material for: Active Video Games for Rehabilitation in Respiratory Conditions: Systematic Review and Meta-Analysis
Source: JMIR Serious Games. 2019 Feb 25;7(1):e10116. doi: 10.2196/10116 (PMC6409512; doi:10.2196/10116)
Supplement: Multimedia Appendix 1 [file games_v7i1e10116_app1.pdf]

Appendix 1: Search strategy

Search terms used for each database on on 30 October 2017.

PubMed

```
(
   "Video games"[mh] OR
   "serious game"[tiab] OR "serious games"[tiab] or "serious gaming"[tiab] OR
   "video game"[tiab] OR "video games"[tiab] OR "video gaming"[tiab] OR
   "virtual game"[tiab] OR "virtual games"[tiab] OR "virtual gaming"[tiab] OR
   "computer game"[tiab] OR "computer games"[tiab] OR "computer gaming"[tiab]
OR
   "digital game"[tiab] OR "digital games"[tiab] OR "digital gaming"[tiab] OR
   "mobile game"[tiab] OR "mobile games"[tiab] OR "mobile gaming"[tiab] OR
   videogame[tiab] OR videogames[tiab] OR videogaming[tiab] OR
   exergame[tiab] OR exergames[tiab] OR exergaming[tiab] OR
   gamification[tiab] OR gamified[tiab] OR gamify[tiab] OR
   xbox[tiab] OR nintendo[tiab] OR
   ( console[tiab] OR "virtual reality"[tiab] AND (game[tiab] OR games[tiab] OR
gaming[tiab]) )

)
AND
(
   "Respiratory Tract Diseases"[mh] OR
   lung[tiab] OR respiratory[tiab] OR pulmonary[tiab] OR
   airway[tiab] OR alveol*[tiab] OR apnea*[tiab] OR apnoea*[tiab] OR
   "cystic fibrosis"[tiab] OR asthma*[tiab] OR antiasthma*[tiab] OR COPD[tiab] OR
   bronchi*[tiab] OR bronchospas*[tiab] OR bronchoconstrict*[tiab] OR
bronchoect*[tiab] OR
   bronchopulmon*[tiab] OR emphysem*[tiab] OR pneumon*[tiab]
)
```

Scopus

```
TITLE-ABS-KEY(
(
      ((serious  OR  video OR  virtual OR computer OR  digital OR mobile) pre/2
(game OR gaming OR games))
      OR videogame OR videogames OR videogaming OR exergame OR exergames
OR exergaming OR
      gamification OR gamified OR gamify  OR
      xbox*  OR  nintendo*  OR
      (console OR "virtual reality"  AND (game OR games OR gaming))
   )
   AND
   (
      lung*  OR  respiratory  OR  pulmonary  OR
      airway  OR  alveol*  OR  apnea*  OR  apnoea*  OR
      "cystic fibrosis"  OR  asthma*  OR  antiasthma*  OR COPD OR
```

```
    bronchi*  OR  bronchospas* OR bronchoconstrict* OR bronchoect* OR
    bronchopulmon*  OR  emphysem*  OR  pneumon*
  )
)
```

Web of Science

```
TS=(
  (
    "serious game"  OR "serious games"  or "serious gaming"  OR
    "video game"  OR "video games"  OR "video gaming"  OR
    "virtual game"  OR "virtual games"  OR "virtual gaming"  OR
    "computer game"  OR "computer games"  OR "computer gaming"  OR
    "digital game"  OR "digital games"  OR "digital gaming"  OR
    "mobile game"  OR "mobile games"  OR "mobile gaming"  OR
    videogame  OR videogames  OR videogaming  OR
    exergame  OR exergames  OR exergaming  OR
    gamification  OR gamified  OR gamify  OR
    xbox  OR nintendo  OR
    ( console  OR "virtual reality"  AND (game  OR games  OR gaming ) )
  )
  AND
  (
    lung*  OR  respiratory  OR  pulmonary  OR
    airway  OR  alveol*  OR  apnea*  OR  apnoea*  OR
    "cystic fibrosis"  OR  asthma*  OR  antiasthma*  OR COPD OR
    bronchi*  OR  bronchospas* OR bronchoconstrict* OR bronchoect* OR
    bronchopulmon*  OR  emphysem*  OR  pneumon*
  )
)
```

EMBASE

```
(
  'video game'/exp OR
  'serious game':ab,ti OR 'serious games':ab,ti or 'serious gaming':ab,ti OR
  'video game':ab,ti OR 'video games':ab,ti OR 'video gaming':ab,ti OR
  'virtual game':ab,ti OR 'virtual games':ab,ti OR 'virtual gaming':ab,ti OR
  'computer game':ab,ti OR 'computer games':ab,ti OR 'computer gaming':ab,ti OR
  'digital game':ab,ti OR 'digital games':ab,ti OR 'digital gaming':ab,ti OR
  'mobile game':ab,ti OR 'mobile games':ab,ti OR 'mobile gaming':ab,ti OR
  videogame:ab,ti OR videogames:ab,ti OR videogaming:ab,ti OR
  exergame:ab,ti OR exergames:ab,ti OR exergaming:ab,ti OR
  gamification:ab,ti OR gamified:ab,ti OR gamify:ab,ti OR
  xbox:ab,ti OR nintendo:ab,ti OR
  ( console:ab,ti OR 'virtual reality':ab,ti AND (game:ab,ti OR games:ab,ti OR
gaming:ab,ti) )

)
AND
(
  'respiratory tract disease'/exp OR
```

```
    lung:ab,ti OR respiratory:ab,ti OR pulmonary:ab,ti OR
    airway:ab,ti OR alveol*:ab,ti OR apnea*:ab,ti OR apnoea*:ab,ti OR
    'cystic fibrosis':ab,ti OR asthma*:ab,ti OR antiasthma*:ab,ti OR COPD:ab,ti OR
    bronchi*:ab,ti OR bronchospas*:ab,ti OR bronchoconstrict*:ab,ti OR
bronchoect*:ab,ti OR
    bronchopulmon*:ab,ti OR emphysem*:ab,ti OR pneumon*:ab,ti
)
```

CINAHL

```
(
    ([MH] "video games") OR TX
    "serious game"  OR TX "serious games"  OR TX "serious gaming"  OR TX
    "video game"  OR TX "video games"  OR TX "video gaming"  OR TX
    "virtual game"  OR TX "virtual games"  OR TX "virtual gaming"  OR TX
    "computer game"  OR TX "computer games"  OR TX "computer gaming"  OR TX
    "digital game"  OR TX "digital games"  OR TX "digital gaming"  OR TX
    "mobile game"  OR TX "mobile games"  OR TX "mobile gaming"  OR TX
    videogame  OR TX videogames  OR TX videogaming  OR TX
    exergame  OR TX exergames  OR TX exergaming  OR TX
    gamification  OR TX gamified  OR TX gamify  OR TX
    xbox  OR TX nintendo  OR TX
    (TX console  OR TX "virtual reality"  AND (TX game  OR TX games  OR TX gaming)
)
)
AND
(
    (MH "Respiratory Tract Diseases") OR TX
    lung  OR TX respiratory  OR TX pulmonary  OR TX
    airway  OR TX alveol*  OR TX apnea*  OR TX apnoea*  OR TX
    "cystic fibrosis"  OR TX asthma*  OR TX antiasthma*  OR TX COPD  OR TX
    bronchi*  OR TX bronchospas*  OR TX bronchoconstrict*  OR TX bronchoect*  OR
TX
    bronchopulmon*  OR TX emphysem*  OR TX pneumon*
)
```

IEEE Xplore

```
(
  "Index Terms":.QT.Video games.QT. OR "Index Terms":Games OR "Metadata":game
OR "Metadata":gamification
)
AND
(
  "Index Terms":lungs OR "Metadata":lung* OR "Metadata":respiratory OR
  "Metadata":pulmonary OR "Metadata":airway OR "Metadata":bronchi* OR
  "Metadata":broncho* OR "Metadata":.QT.cystic fibrosis.QT. OR "Metadata":asthma*
)
```
