# Supplementary material for: Active Video Games for Rehabilitation in Respiratory Conditions: Systematic Review and Meta-Analysis
Source: JMIR Serious Games. 2019 Feb 25;7(1):e10116. doi: 10.2196/10116 (PMC6409512; doi:10.2196/10116)
Supplement: Multimedia Appendix 2 [file games_v7i1e10116_app2.zip › template_manuscript.docx]

**Systematic review and meta-analysis**

**Title:** Active video games for rehabilitation in respiratory conditions: a systematic review and meta-analysis

**Authors**
Joshua Simmich (M. Phty St.)
Centre for Research Excellence in Telehealth, University of Queensland, Brisbane, Australia
School of Health and Rehabilitation Sciences, University of Queensland, Brisbane, Australia

Anthony J. Deacon (MBBS, M. IT, M. Bus)
Centre for Research Excellence in Telehealth, University of Queensland, Brisbane, Australia
School of Electrical Engineering and Computer Science, Queensland University of Technology, Brisbane, Australia

Trevor G. Russell (M. Phty, PhD)
Centre for Research Excellence in Telehealth, University of Queensland, Brisbane, Australia
School of Health and Rehabilitation Sciences, University of Queensland, Brisbane, Australia

**Corresponding author:** Joshua Simmich, School of Health and Rehabilitation Sciences, University of Queensland, Brisbane, 4072, Australia. Email: [joshua.simmich@uqconnect.edu.au](mailto:joshua.simmich@uqconnect.edu.au)

**Author contributions:** Joshua Simmich (Literature search, data collection, study design, analysis of data, manuscript preparation, review of manuscript), Anthony Deacon (data collection, analysis of data, manuscript preparation, review of manuscript), Trevor Russell (study design, manuscript preparation, review of manuscript)

**Research location:** University of Queensland, Brisbane, Australia

**Funding and conflict of interest statement:** This research did not receive any specific grant from funding agencies in the public, commercial or not-for-profit sectors. The authors have no conflicts of interest to declare.

**Keywords:** video games; exercise; physical activity; COPD; cystic fibrosis; asthma

**Abbreviations:** AVG = active video games, DSL = DerSimonian-Laird method, HKSJ = Hartung-Knapp-Sidik-Jonkman method, HR = heart rate

# Abstract

**Background**: Exercise and physical activity are key components of treatment for chronic respiratory diseases. However, the level of physical activity and adherence to exercise programs is low in people with these diseases. Active video games (AVG) may provide a more engaging alternative to traditional forms of exercise.

**Objective**: This review examines the effectiveness of game-based interventions on physiological outcome measures, as well as adherence and enjoyment, in subjects with chronic respiratory diseases.

**Methods**: A systematic search of the literature was conducted, with full-texts and abstracts included where they involved an AVG intervention for participants diagnosed with respiratory conditions. A narrative synthesis of included studies was performed. In addition, meta-analysis comparing AVGs to traditional exercise was undertaken for four outcome measures: mean heart rate (HR) during exercise, peripheral blood oxygen saturation (S_pO2_) during exercise, dyspnoea induced by the exercise and enjoyment of the exercise.

**Results**: A total of 13 full-text articles corresponding to 12 studies were included in the review.

Interventions predominantly used games released for the Nintendo Wii^TM^ (eight studies) and Microsoft Xbox Kinect^TM^ (three studies). There were five studies which examined acute effects of a single session of AVGs, and seven studies which examined long-term effects after multiple sessions of AVGs. Trials conducted over more than one session varied in duration between 3 and 12 weeks. In these, AVG interventions were associated with either similar or slightly greater improvements in outcomes such as exercise capacity when compared a traditional exercise control and also generally demonstrated improvements over baseline or non-intervention comparators. There were few studies of unsupervised AVG interventions, but these reported adherence was high and maintained throughout the intervention period. In addition, AVGs were generally reported to be well-liked and considered feasible by participants.

For outcome measures measured during a single exercise session, there was no significant difference between AVGs and traditional exercise for HR (mean difference=1.44 beats per minute, 95% CI (confidence interval) [-14.31, 17.18]), S_pO2_ (mean difference=1.12 percentage points, 95% CI [-1.91, 4.16]) and dyspnoea (mean difference=0.43 Borg units, 95% CI [-0.79, 1.66]), but AVGs were significantly more enjoyable than traditional exercise (Hedges’ *g* standardised mean difference=1.36, (95% CI [0.04, 2.68]).

**Conclusions**: This review provides evidence that AVG interventions, undertaken for several weeks, can provide similar or greater improvements in exercise capacity and other outcomes as traditional exercise. Within a single session of cardiovascular exercise, AVGs can evoke similar physiological responses as traditional exercise modalities but are more enjoyable to subjects with chronic respiratory diseases. However, there is very limited evidence for adherence and effectiveness in long-term unsupervised trials, which should be the focus of future research.

# Introduction

Chronic respiratory diseases such as asthma [1], chronic obstructive pulmonary disease (COPD) [2] and cystic fibrosis (CF) [3] represent a significant disease burden globally, accounting for approximately 7% of all global deaths [4]. Recent systematic reviews have shown physical activity and exercise to be beneficial for pulmonary function and health-related quality of life in both asthma [5] and CF [6]. Physical activity is also negatively associated with mortality and exacerbations in COPD [7]. Exercise is a key component of pulmonary rehabilitation for COPD, which a recent systematic review has shown to improve quality of life and exercise capacity in subjects [8]. Exercise-based pulmonary rehabilitation can also be an effective intervention for other respiratory conditions [9,10]. Exercise is prescribed in clinical guidelines for both CF [11] (grade B recommendation) and COPD [12] (strong recommendation).

Physical activity is defined as any bodily movement resulting in energy expenditure, and exercise is a subset of physical activity that is planned, structured, repetitive and aimed at improving or maintaining physical fitness [13]. Despite the clear evidence for physical activity and exercise in the management of respiratory conditions, adherence in these populations is generally low. Objective measurements indicate that almost half of adults with CF do not achieve 30 minutes of physical activity per day [14]. Similarly in COPD, pulmonary rehabilitation has attendance and completion rates as low as 70% and 40% respectively [15,16], and a recent meta-analysis has shown people with COPD are generally severely physically inactive [17]. Low motivation has been identified as a key internal barrier to physical activity in asthma [18], COPD [19] and CF [20], and enjoyment of physical activity has been shown to be a key motivator of exercise in these populations [20–22].

Video games have attracted attention as a novel way of delivering healthcare interventions with the potential to motivate change in health behaviours [23]. Video games can be designed to incorporate a physical activity or exercise component, and these are known as active video games (AVGs) or exergames [24]. The use of AVGs has been demonstrated in many age groups and clinical conditions as an enjoyable form of rehabilitation [25,26], and can also be designed to encompass other treatments like airway clearance or spirometry [27–29]. Games used in rehabilitation may either be designed specifically for this purpose or be commercial games designed for recreational exercise that have been adapted by clinicians and researchers for use in rehabilitation.

Reviews of the use of games for purposes such as improving balance in the elderly, rehabilitation of motor function in stroke or rehabilitation of upper limb functioning in cerebral palsy, have found small effects in favour of games over traditional therapies [25,26]. Several advantages of video games over traditional rehabilitation have been posited, such as objective measurement of performance and progress (e.g. time spent playing the game, number of repetitions performed), low cost and improved motivation and adherence [30]. The results of individual studies have reported that a single session of an AVG was considered more enjoyable than traditional exercise in subjects with CF [31,32] and COPD [33], in keeping with a systematic review which concluded that AVGs are considered enjoyable by many different populations of subjects undergoing rehabilitation [26]. Given the significant positive correlation between emotional judgment (such as enjoyment) of physical activity and frequency of physical activity in healthy adults [34,35], it is likely that interventions which improve enjoyment of exercise will improve adherence to exercise. In healthy populations, studies have found a correlation between enjoyment of an active video game and exercise adherence [36] or energy expenditure during play [37]. However, the relationship between games, enjoyment, adherence and overall effectiveness are poorly understood, especially in chronic respiratory conditions.

To date only one systematic review specifically examining the use of video games in respiratory conditions exists. This review was limited in scope as it only aimed to review the exercise intensity of AVGs for people with CF [38], and therefore only included five studies. Another broad review of video games in rehabilitation did not include respiratory conditions, and instead focused on ageing and pathologies such as stroke and Parkinson’s disease for which there was more evidence [39]. Other broad reviews have focused only on pathologies for which there are randomised controlled trials looking at changes in rehabilitation outcomes over time [25] or only on games aimed at improving motor function rather than cardiorespiratory function [26]. Reviews that have included respiratory conditions have been limited to scoping reviews rather than systematic reviews [40,41].

By comparing multiple diseases, this review provides a broader picture of the use of video games for rehabilitation in respiratory conditions than previous reviews. Also, it examines evidence more specific to the posited advantages of video games in rehabilitation, by investigating not only clinical effectiveness of video games but also their economic feasibility and reported measures of adherence and enjoyment. In addition, it should provide clinicians and researchers with a unified view of the literature on this newly emerging field of game-based pulmonary rehabilitation. a. The primary aim of this review is to evaluate the effectiveness of AVGs for three categories of outcomes: a) clinical outcomes, b) economic feasibility, and c) patient enjoyment and adherence. The secondary aim is to compare AVGs to traditional exercise for the afore-mentioned outcome measures. This review will examine the hypotheses that 1) AVGs improve clinical outcomes, are economically feasible and enjoyable; and 2) that AVGs produce equivalent clinical outcomes as traditional exercise programs, but are more cost-effective, more enjoyable and have higher adherence.

# Methods

## Search strategy

Studies were identified by searching the following electronic databases: PubMed, Scopus, Web of Science, EMBASE, CINAHL and IEEE Xplore. Medical subject headings (MeSH) terms and equivalent subject headings were used for PubMed, EMBASE, CINAHL and IEEE Xplore. Search terms for active video games (e.g. ‘video games’, ‘computer games’, ‘digital games’, ‘gamification’ etc), were combined with terms for respiratory diseases (e.g. ‘lung’, ‘pulmonary’, ‘respiratory’, ‘airway’, ‘cystic fibrosis’, ‘COPD’, etc) derived from key terms for respiratory diseases listed in the Cochrane Airways Group Search Strategies document [42]. For IEEE Xplore broader search terms were found be suitable. There were no date or language restrictions placed on the search, though all search terms were in English. Full details of the final search strategies for each database are available in Multimedia Appendix 1.

The search was completed on 30 October 2017, and each database was searched from inception to the date of search. Search results were imported into Zotero (Version 4.0, George Mason University, Fairfax, VA, USA) and EndNote (Version 18.0, Clarivate Analytics, Philadelphia, PA, USA) to automatically remove duplicate citations, and results were further searched by hand to remove remaining duplicates. The combined list of citations was then imported into Covidence online review manager (Veritas Health Innovation, Melbourne, VIC, Australia) for screening of titles, abstracts and full-texts. Hand searches of reference lists and citation searches for literature citing included studies were also conducted.

## Study selection

Inclusion and exclusion criteria are outlined in Textbox 1 and Textbox 2. Two reviewers (J.S., A.D.) independently screened the titles and abstracts of identified articles according to inclusion and exclusion criteria, and potentially relevant articles were identified for full-text screening. Full-text articles were retrieved and screened independently by the same two reviewers, and reasons were given for all excluded citations. In both stages of screening, disagreements were resolved by consensus or in consultation with a third reviewer (T.R.) if disagreement persisted.

| Inclusion criteria |
| --- |
| 1) Is an experimental (or quasi-experimental) study of an intervention including quantitative data. This includes studies of the following designs: Randomised controlled trials, quasi-randomised trials, interrupted time series, controlled before-and after studies, uncontrolled before-and-after studies |
| 2) Participants are predominantly people diagnosed with respiratory conditions. Respiratory conditions include any disease under the National Library of Medicine Medical Subject Headings (NLM MeSH) term “Respiratory Tract Diseases”[C08], including lung cancer [C08.381.540], cystic fibrosis[C08.381.187], asthma[C08.127.108] and chronic obstructive pulmonary disease[C08.381.495.389]. |
| 3) Includes an intervention involving any form of video game, console game, virtual reality game or gamified mobile/computer app that requires players to perform a physical action that forms part of their rehabilitation (such as perform cardiovascular exercise, use a spirometer, perform airway-clearance techniques, etc) |

| Exclusion criteria |
| --- |
| 1) Study is not experimental or does not include quantitative data. Therefore, publications of the following designs are excluded: descriptive and observational studies (case series, case-control, cohort studies, qualitative research designs, etc), secondary research studies (reviews, meta-analyses) and protocol papers |
| 2) Includes only interventions that do not require participants to physically perform rehabilitative actions in order to progress in the game, such as educational games that merely encourage (or teach the importance of) consistent medication use or physical activity |
| 3) Is not a full-text article published in a peer-reviewed journal) |

## Data extraction and quality assessment

One reviewer (J.S.) extracted data and assessed the quality of each included study. The extracted data and the results of quality assessment were then independently checked by a second reviewer (A.D.) for accuracy and completeness. Quality was assessed using the ‘Checklist for Measuring Quality’ assessment tool by Downs and Black [43].

Where multiple articles reported data from the same study and cohort, these were considered as one study for the purposes of data extraction and quality assessment.

## Data synthesis

Only studies that compared AVGs to a control exercise (rather than an exercise test) were included in the meta-analysis (quantitative synthesis), and only for outcome measures reported at similar time points in at least three different studies. A narrative synthesis was undertaken for all other studies.

A random effects model was used to assess all outcome measures. The method of Hartung-Knapp-Sidik-Jonkman (HKSJ) [44,45] was employed. This method is recommended over the more common DerSimonian and Laird (DSL) method [46,47] for meta-analyses where only few studies are available (the DSL method was also calculated for comparison). Pooled effect sizes were reported as weighted mean differences (MD) between treatments in the original units when possible, with a 95% confidence interval (CI). If studies used different scales, effect sizes were expressed as standardised mean differences (SMD) using Hedges’ *g* [48], also with a 95% CI. Statistical heterogeneity was estimated using the point estimate and 95% confidence interval of the *I*^2^ statistic.

Additionally, 90% confidence intervals were calculated as above for those outcome measures where included studies attempted to demonstrate equivalence between interventions (heart rate, dyspnoea and peripheral blood oxygen saturation). To demonstrate equivalence with α=0.05, the 90% confidence interval must lie between the pre-determined equivalence margins [49]. For HR evoked during exercise we used a difference of 10 beats per minute (bpm) (i.e. -10 to +10bpm) for our equivalence margins as there is no agreed-upon minimum important difference and two previous studies nominated a difference of 10bpm as important when performing their initial sample size calculations [31,32]. For dyspnoea, equivalence margins of 1 unit (i.e. -1 to +1) were used, as it has been suggested that the minimum clinically important difference for the modified 11-point (0-10) Borg scale is 1 unit [50]. For peripheral blood oxygen saturation (S_pO2_), a drop of 4% or more has been used as a definition for exertional oxygen desaturation in CF [51,52] and COPD [53], so the width of our equivalence margins was set at 4% (i.e. -2 to 2%).

Data from cross-over trials were treated as paired data in the meta-analysis where raw mean differences could be calculated. An estimate of the within-subject standard deviation was estimated where possible from the reported confidence intervals of the difference between treatments (or *P*-values from a paired *t*-test of the difference between treatments). From the within-subject standard deviation a within-subject correlation was calculated using methods outlined by Elbourne et al. [54]. Where a within-subject correlation could not be calculated, the correlation from another study of similar design was used as an estimate. A sensitivity analysis, where this correlation was altered to either 0 (uncorrelated) or 1 (perfectly correlated), was conducted to test the influence this assumption had on the pooled estimate. Where results had to be reported as standardised mean differences, due to comparing across different scales, data from cross-over trials was treated as if it came from independent groups.

Data reported only as median and inter-quartile range were converted to mean and standard deviation using formulae from Wan et al. [55]. Ordinal data, such as that from Likert or Likert-type items, was treated as interval data for the purposes of this meta-analysis. Authors of the studies included in the meta-analysis were contacted for additional information, and when provided this additional data was used in place of the summary data reported in the publications.

Data conversions and statistical analysis were performed using R software (Version 3.4.0, R Foundation for Statistical Computing, Vienna, Austria), with meta-analysis using the “metafor” package, version 1.9-9 [56].

Code used to generate the analysis and sections of the article was written as a dynamic manuscript, using the R package “rmarkdown” version 1.3 [57], and is available in Multimedia Appendix 2 for reproducing all data conversions and statistical analysis. Data used in this manuscript are located in Multimedia Appendix 3 and Multimedia Appendix 4.

# Results

|[INSERT FIGURE 1 ABOUT HERE]|

Figure 1 shows the PRISMA flow diagram [58] which illustrates the screening and selection process. The initial literature search gave a total of 1040 results, with 612 unique records remaining after duplicates were removed (PRISMA flow diagram [58] shown in Figure 1). After screening titles and abstracts, 91 results that met the inclusion criteria remained for full-text assessment. A total of 13 articles passed both the inclusion and exclusion criteria after full-text assessment and were included in the narrative synthesis, which corresponded to 12 studies. The interventions and outcomes studied were heterogeneous, with the meta-analysis including only four studies for heart rate (HR), three studies for mean peripheral blood oxygen saturation (S_pO2_), three studies for reported dyspnoea and three studies for enjoyment for the exercise interventions. Manual screening of titles listed in the reference lists of included full-text articles did not yield any additional articles. One potential article was found by citation tracking but did not pass full-text screening.

## Study characteristics

There were a total of 292 participants in the 12 full-text articles. Sample sizes ranged from a small observational study of 7 to larger studies with 40 participants (parallel-groups design), 30 participants (crossover-trial) and 32 participants (observational study). In total, six studies included participants with CF [27,31,32,59–61], three studies included participants with COPD [33,62,63] and three with other or mixed respiratory conditions [64–66]. Ages were varied due to the respiratory conditions of interest: studies of asthma included only children, studies of CF included children or young adults, and studies of lung cancer and COPD included only older adults. The randomised experimental studies often used crossover designs [27,31–33,59] with only three studies [60,64,66] employing a two parallel groups design. Non-randomised and observational studies represented the remaining four studies [61–63,65]. Additional details about each included study, including the specific games played on each device, can be found in Multimedia Appendix 5.

Interventions were predominantly commercially available off-the-shelf AVGs, namely games released for the Nintendo Wii^TM^ and Microsoft Xbox Kinect^TM^. The Wii^TM^ was used in eight of 12 studies [31,33,59,60,62,63,65,66], whereas the Kinect^TM^ was used in three of 12 studies [32,61,64]. Only one study reported the testing of custom-designed hardware and games for respiratory conditions [27].

Four of 12 included studies reported randomised studies examining the longer-term effects of AVGs versus traditional training in respiratory conditions [27,60,64,66]. One examined the effect of AVGs versus treadmill training in asthma, with training sessions twice a week over eight weeks [64]. Another examined the effect of AVGs in combination with usual pulmonary rehabilitation compared to usual pulmonary rehabilitation alone, with three weeks of pulmonary rehabilitation in both groups but one week of AVGs in the final week of the experimental group [66]. A third compared a custom spirometer game to control device over a 2-3 week period, with participants prompted but not required to use the device [27]. There were also three studies [62,63,65] that looked at longer-term effects of AVGs but which were not randomised and/or controlled experiments.

There were five studies [31–33,59,61] which examined acute effects of a single session of AVGs. For most of these the active video game was compared to traditional exercises [31–33,64], though some compared AVGs to maximal [61] or submaximal exercise tests [59].

## Quality assessment

Multimedia Appendix 6 describes the quality of the included studies as assessed on the Downs and Black checklist.

Due to the nature of the intervention, no study blinded participants to treatment and only three blinded those assessing all outcome measures [27,60,64]. Additionally, none attempted to demonstrate their participants were representative of the population or recruited their participants randomly. Only four studies reported concealing patient allocation until the experiment was complete [31,32,60,64]. Only six studies reported adequate statistical power [31,32,60,61,64,66], though one study did perform a power calculation but was underpowered due to dropouts [62].

## Meta-analysis

A meta-analysis comparing AVGs to traditional exercise was only able to be conducted for four outcome measures: mean heart rate (HR) during exercise, peripheral blood oxygen saturation (S_pO2_) during exercise, dyspnoea induced by the exercise and enjoyment of the exercise.

|[INSERT FIGURE 2 ABOUT HERE]|

### Mean heart rate (HR)

The mean HR of AVGs and traditional exercise was assessed in four studies with a total of 95 participants. As shown in Figure 2A, the mean difference between mean HR during AVGs and mean HR during traditional exercise was 1.44 (95% CI [-14.31, 17.18]) bpm, indicating there was no significant difference between the two interventions. Statistical heterogeneity by *I*^2^ was found to be 93% (95% CI [79%,99%]).

The lower and upper bounds of the 90% CI for the difference in HR between interventions are -10.21bpm and 13.08bpm respectively, which are not within the equivalence margins of -10bpm and 10bpm. Therefore these treatments cannot be considered equivalent at the α=0.05 level.

### Mean peripheral blood oxygen saturation (S_pO2_)

The mean S_pO2_ of AVGs and traditional exercise was assessed in three studies with a total of 59 participants. As shown in Figure 2B, the mean difference between mean S_pO2_ during AVGs and mean S_pO2_ during traditional exercise was 1.12 (95% CI [-1.91, 4.16]) raw percentage points, indicating there was no significant difference between the two interventions. Statistical heterogeneity by *I*^2^ was found to be 93% (95% CI [74%,100%]).

The lower and upper bounds of the 90% CI for the difference in mean S_pO2_ between interventions are -0.94% and 3.18% respectively, which are not within the equivalence margins of -2% and 2%. Therefore these treatments cannot be considered equivalent at the α=0.05 level.

### Dyspnoea

The mean dyspnoea of AVGs and traditional exercise was assessed in three studies with a total of 59 participants. As shown in Figure 2C, the mean difference between mean reported dyspnoea during AVGs and during traditional exercise was 0.43 (95% CI [-0.79, 1.66]) points on the modified 11-point (0-10) Borg scale, indicating there was no significant difference between the two interventions. Statistical heterogeneity by *I*^2^ was found to be 81% (95% CI [18%,100%]).

The lower and upper bounds of the 90% CI for the difference in reported dyspnoea between interventions are -0.4 and 1.27 points on the modified 11-point (0-10) Borg scale respectively, which are not within the equivalence margins of -1 and +1 points. Therefore these treatments cannot be considered equivalent at the α=0.05 level.

### Enjoyment

Measures of enjoyment of AVGs and traditional exercise were reported in three studies with a total of 59 participants. As shown in Figure 2D, the standardised mean difference between mean reported enjoyment during AVGs and during traditional exercise was 1.36 (95% CI [0.04, 2.68]), using Hedges *g*, indicating there was a significant difference between the two interventions. Statistical heterogeneity by *I*^2^ was found to be 47% (95% CI [0%,99%]).

In the absence of other information, Cohen proposed that standardised effect sizes (such as Cohen’s *d* or Hedges’s *g*) of ≥0.8 be considered as large effects [67]. The effect of enjoyment of AVGs observed in the present meta-analysis would therefore be considered a large effect.

### Sensitivity analysis

A sensitivity analysis was conducted by altering the correlation coefficients of cross-over studies where the correlation could not be calculated, which had been assumed to be equal to the correlation calculated in another similar study. This assumption had negligible influence on the outcomes measures of HR and enjoyment, and little influence on the results of dyspnoea (see Multimedia Appendix 7 for full analysis).

## Narrative synthesis

### Active video games vs exercise test protocols

Results from two studies examining the physiological responses to a single session of active video gaming compared to a maximal exercise tests (e.g. cardiopulmonary exercise testing, incremental shuttle walk test) suggest that video games usually provide a less intense exercise stimulus than a maximal exercise text. Holmes et al. [61] compared the intensity of exercise elicited from Microsoft Xbox Kinect^TM^ to the maximal exercise capacity of participants with CF, and found the average HR achieved during the 10 minute video game session was 86% (95% CI [81, 92]) of the peak HR achieved during a cardiopulmonary exercise test (using a modified Godfrey Cycle Ergometer Protocol). From this they conclude the game represented an exercise intensity of 6.1±1.8 metabolic equivalents (METS), just above the 6METS threshold for vigorous intensity activity in healthy adults [68]. They also found the game produced lower reported dyspnoea, lower rating of perceived exertion (RPE) and caused less oxygen desaturation than the maximal exercise test.

Another study compared AVGs to a submaximal exercise test, such as a 6-minute walk test (6MWT). Del Corral et al. (2014) [59] performed a randomised cross-over observational trial to compare the physiological responses to five minutes of three different AVGs using the Nintendo Wii^TM^ console and a 6MWT (repeated twice). They found the the two games that were focused on aerobic exercise (*EA Sports Active*^TM^ and *Family Trainer Extreme Challenge*) both elicited higher VO_2_ than the 6MWT. However, the game that included balance exercises in addition to aerobic exercises (*Wii Fit*^TM^ *Plus*) elicited a lower volume of oxygen consumption (VO_2_) during the final three minutes of exercise than the 6MWT. HR was also lower in the *Wii Fit*^TM^ *Plus* game than the 6MWT but did not differ between the 6MWT and the other two games, and there was no statistically significant difference in dyspnoea between any intervention.

### Active video games vs rest only

One study compared physiological responses to AVGs to rest without comparison to a control exercise. The average response to several Nintendo Wii^TM^ gaming sessions in subjects with COPD was presented in Wardini et al. , showing that games significantly increased HR (by a mean of 13.8bpm) and dyspnoea, and decreased S_pO2_, compared to rest.

### Long-term physiological effects of active video games

In total, seven studies included more than one session of an AVG intervention.

When compared to those seen in a control intervention, game interventions were associated with either similar or slightly greater improvements in measured outcomes. For example, in a randomised controlled trial with parallel groups Gomes et al. [64] reported both Microsoft Xbox Kinect^TM^ and treadmill training for eight weeks improved aerobic capacity and asthma control but only the game intervention showed a statistically significant improvement in exhaled fraction of nitric oxide (FeNO). Likewise, Bingham et al. [27] reported forced expiratory volume in 1 second (FEV1) was better maintained during the game intervention than the control intervention. Mazzoleni et al. [66] reported both 6MWT distance and dyspnoea improved more in the participants performing pulmonary rehabilitation with an adjunct AVG than pulmonary rehabilitation alone.

Active video game interventions generally demonstrated improvements over baseline or non-intervention comparators. In a randomised 6 week trial, del Corral et al. [60] reported the Nintendo Wii^TM^ group, but not the control group receiving no intervention, improved both modified shuttle walk test (MSWT) and 6MWT distances, as well as several measures of strength. Likewise in a single-arm study, Albores et al. (2013) [62] reported a statistically significant improvement in endurance shuttle walk test (ESWT), number of sit-to-stands in 30s and number of arm lifts in 30 seconds after 12 weeks of unsupervised Nintendo Wii^TM^ training in subjects with COPD. Similarly in another single-arm study, Hoffman et al. (2013) [65] reported an increase in functional performance, as measured by number of steps per day, during the game intervention period. No studies reported AVGs were inferior to control or were associated with a negative change to an outcome measure relative to baseline. Wardini et al. [63] performed a 3-4 week intervention but did not report changes in outcome measures over time.

### Adherence and patient preference

Five studies examined adherence to unsupervised active video game treatments, and generally reported adherence was higher than 70% and maintained throughout the intervention period [27,60,62,63,65]. The controlled trial by del Corral [60] reported the adherence to the home intervention was 95% during the 6 week trial, but adherence dropped in the 12-month followup with 65% no longer using the AVG at all. A single-arm study Hoffman et al. (2013) [65] reported a rate of adherence of 96.6±3.4% to a Nintendo Wii^TM^ intervention in subjects who had undergone surgery for suspected lung cancer. Also, the mean time spent performing walking exercise with the Wii^TM^ increased during the first six weeks post-surgical recovery period and was maintained for the following 10 weeks [69]. In their uncontrolled (single-arm) study Albores et al. (2013) [62] instructed subjects with COPD to use a Nintendo Wii^TM^ for exercise for at least 30 minutes on most days of the week. Subjects self-reported exercising at the prescribed frequency and duration throughout the 12 week intervention, with a frequency of 5.7±1.0 days per week and a weekly total of 3.0±0.9 hours. Bingham et al. [27] did not prescribe the use of their intervention, so adherence could not be calculated. Nonetheless, their game intervention was used for significantly more minutes per day than the control (4.8±4.4 vs 1.6±1.8), with no difference in the number of days each intervention was used. In contrast, in a study by Wardini et al. [63] the attendance rate to the game intervention (64±35%) was lower than for the pulmonary rehabilitation program (88±13%), though the game was delivered as an adjunct rather than an alternative program and no statistical comparison was performed.

Measures of patient preferences, likeability or perceived feasibility were measured in several studies (in addition to enjoyment, which was summarised in the meta-analysis), showing that games were generally considered likeable and feasible interventions [31,33,63,66]. For example, Kuys et al. [31] reported that both the Nintendo Wii^TM^ intervention and the treadmill or bicycle control exercise were rated highly effective and feasible without significantly differences between the two interventions. Similarly, LeGear et al. [33] reported similar responses between game exercise and treadmill exercise for perceived safety and whether the participants could see themselves performing that exercise at home. Using a 7-item, 7-level Likert-type scale, Mazzoleni et al. [66] reported no significant difference between the acceptability of pulmonary rehabilitation alone (43.9±3.0) or with active video gaming as an adjunct (42.4±3.5). Wardini et al. [63] reported the overall enjoyment of the adjunct game intervention to be 8.0±2.6cm on a 10cm visual analogue scale. Also, participants gave a mean score of 8.0±2.6cm when asked if they would recommend the adjunct program to another patient with COPD and a rating of 7.0±3.7cm when asked if they would consider purchasing a game system of their own.

### Adverse events

No studies reported the occurrence of adverse events linked to AVGs. Wardini et al. [63] reported one patient with COPD and coronary artery disease requiring the use of nitroglycerin, and 5 subjects experiencing transient but asymptomatic oxygen desaturation below 85%, but did not report whether these were a result of the active video game intervention or the control intervention.

### Economic analysis

No studies performed an economic analysis comparing AVGs to alternative forms of exercise or rehabilitation.

# Discussion

This systematic review aimed to examine the hypotheses that 1) AVGs improve clinical outcomes, are economically feasible and enjoyable; and 2) that AVGs produce equivalent clinical outcomes as traditional exercise programs, but are more cost-effective, more enjoyable and have higher adherence. AVGs generally demonstrated improvements in exercise capacity or functional performance over baseline or non-intervention comparators, and were generally reported to be well-liked by participants. There were no differences between AVGs in physiological outcomes when compared to traditional exercises (such as treadmill or stationary cycling) in the laboratory over a single session, but AVGs were enjoyed more by participants. Despite few long-term trials, the limited evidence suggests AVGs may provide similar or greater effects on clinical outcome measures compared to traditional forms of exercise. None of the included studies provided any evidence for the economic feasibility of the interventions.

The results of the present review corroborate the findings of a systematic review into the effects of AVGs on people with CF [38], which found the heart rate (HR) response to active video gaming was similar to the traditional methods of physical training. One of the studies included in that review was excluded from the present review as it did not compare AVGs to traditional exercise or to rest [70]. This study found the HR target for moderate physical activity (64% of predicted maximum HR) was met during the boxing activity of the Nintendo Wii^TM^ game *Wii Sports*^TM^ and exceeded by the free jogging activity in the Nintendo Wii^TM^ game *Wii Fit*^TM^ in children with CF.

Likewise, the results of the present review are consistent with a meta-analysis of AVGs for healthy adults and children by Peng et al. [71], who concluded that the HR, energy expenditure and oxygen consumption were no different between AVGs and traditional physical activities. In contrast, a more recent meta-analysis on AVGs in healthy children [72] found that AVGs had a large positive mean effect on HR, small positive mean effect size on enjoyment and a similar rate of perceived exertion compared to traditional exercise in a laboratory setting.

## Physiological responses to active video games

The results of the meta-analysis indicate that the physiological response to AVGs was not significantly different from traditional exercises (Figure 2). However, statistical equivalence was not demonstrated for any of these physiological outcomes, possibly due to there being so few included studies and the heterogeneity between studies.

The physiological response to AVGs may partly derive from psychological arousal rather than the physical workload of the activity. An increase in HR and respiratory rate has been observed in healthy adults and children playing inactive video games with traditional controllers [73,74]. Likewise, Sherman et al. [75] found the HR changes were similar whether the game was controlled with a joystick or a motion sensor detecting the movement of the player’s body. However, the HR changes reported in the studies included in the present review are greater than those seen in response to inactive video games. Additionally, the pooled effect of AVGs on HR and dyspnoea was no different from traditional moderate intensity exercise, so psychological arousal likely had only a small or negligible contribution to the observed physiological responses.

Further, in each study included in the meta-analysis the participants were instructed to perform both AVGs and traditional exercises at similar self-perceived intensities and supervised to ensure compliance. For example, in both Kuys et al. [31] and Salonini et al. [32] participants in each intervention were instructed to exercise at an intensity corresponding to 3-5 on the modified Borg scale. Therefore it is not known whether these interventions would produce similar workloads in a situation where subjects were unsupervised or allowed to exercise at a self-selected intensity.

Additionally, one included study [32] did not use the 11-point (0-10) modified Borg scale used by other included studies but rather a visual analog scale that was also 0-10, though results were nonetheless pooled as if they are equivalent scales. Dyspnoea assessed on a visual analog scale correlates strongly with the modified Borg scale [76,77], but the correlation between these two assessment scales is not perfect so may have been a source of additional variability. Furthermore, Salonini et al. [32] assessed dyspnoea during exercise, whereas other studies [31,33] assessed dyspnoea at the end of the exercise bout. Both of these differences in assessment between studies may have further contributed to the heterogeneity observed in the meta-analysis.

Additionally, the present meta-analysis pooled values for mean S_pO2_ as this was the most commonly reported measure, but minimum S_pO2_ may be more relevant to detecting the occurrence of exertional oxygen desaturation that can occur in CF [51,52] and COPD [53,78]. Only two papers reported minimum S_pO2_. Kuys et al. [31] found minimum S_pO2_ was 2% lower in traditional exercise compared to game-based exercise but this was not a significant difference (95% CI [-2,6]). LeGear et al. [33] reported minimum S_pO2_ was 91.3% during the game vs 88.7% in the treadmill but did not perform a statistical comparison between these interventions (nor report sufficient data to enable one to be calculated).

## Other responses to active video games

The pooled estimate of enjoyment of AVGs was significantly higher than traditional exercises, however this estimate was based upon a small number of studies with heterogeneous outcome measures. Each included study assessed enjoyment using different measures and none used an experimentally validated measure of enjoyment, which limits comparison between included studies and to the literature as a whole. Established, validated instruments for assessing enjoyment of exercise include the Physical Activity Enjoyment Scale (PACES) [79] or Feeling Scale [80]. In healthy sedentary adults, an increase of 1 unit on the 11-point bipolar (-5 to +5) Feeling Scale during moderate intensity treadmill walking is associated with an increase of 15 minutes of weekly physical activity at six month followup [81], though this result was observational and causation therefore couldn’t been demonstrated. Nonetheless, it’s possible that the 2 unit difference on the 0-10cm visual analog scale in response to AVGs compared to traditional exercise reported by Kuys et al. [31], which corresponds with the pooled estimate of the present meta-analysis (as shown in Figure 2D), could result in at least a similar increase in physical activity in subjects with respiratory diseases.

The included studies assessed enjoyment of exercise by assessing participants after exercise rather than during. Enjoyment assessed after exercise may be biased due either to the time between exercise and giving an enjoyment response or to an emotional response to completion of the task.

Some studies used a continuous mode of exercise as a control (continuous cycling or treadmill walking), whereas participants playing AVGs performed periods of exercise interspersed with rest periods between game levels [32,33]. Interval training has been demonstrated to be more enjoyable than continuous exercise in healthy active populations [82,83], which may explain at least part of the enjoyment effect of AVGs over traditional exercise in the included studies. In addition, the video game intervention often involved different exercises and a greater variety of exercises compared to the control [31,33]. Thus, it is possible that a control intervention which also consisted of a variety of exercises (e.g. dancing, boxing and running) may have been just as enjoyable as the active video game. In future, studies should compare AVGs to interval training consisting of a routine or circuit with an equivalent variety of exercises.

In addition, several included studies noted that the greater enjoyment found in AVGs may be because participants found them more novel than traditional exercises. Studies of longer duration must be performed to see if enjoyment is maintained in subjects with respiratory diseases using AVGs as part of their rehabilitation, and if this corresponds to greater adherence to exercise. Most studies used supervised exercise training and so could not assess the effect of video games on adherence to unsupervised home exercise or self-chosen duration of exercise. The greater enjoyment seen in video games could fail to translate to greater adherence or longer durations of exercise, perhaps because the additional technological requirements of AVGs compared to other modes of exercise (such as jogging) may decrease adherence in a real-world situation.

No adverse events linked to the active video game intervention were reported in any included studies, though the small sample sizes of included studies may have limited their ability to detect rare adverse events.

## Strengths and limitations of this review

This review includes evidence from multiple respiratory conditions, which provides a broader picture using a greater quantity of evidence than is currently available for any condition individually. Additionally, despite the different methodologies and reported outcome measures this review transformed and standardised the data as best as possible to enable a meta-analysis to be conducted, using the conservative Hartung-Knapp-Sidik-Jonkman (HKSJ) approach [45,80].

The main limitation of this review is the small number and significant heterogeneity of the included studies. This review included mostly relatively small studies that examined multiple different respiratory diseases, within different age groups and using different interventions (e.g. types of software and technology). A subgroup analysis was not performed for age groups or disease populations as evidence was limited to just one or two studies in each group. This clinical heterogeneity was the reason for performing a narrative synthesis for several outcomes. The statistical heterogeneity was also high, however due to the low number of studies even the estimates of statistical heterogeneity were highly uncertain.

An additional limitation is the inclusion of only studies published in full, which ensured data came only from high-quality studies but may have caused the review to be vulnerable to publication bias, which also may have limited previous reviews [26]. Language bias may also have been present as all search terms were in English, although indexed terms were used which may have minimised potential language bias. Finally, the HKSJ estimation method, though chosen because it provides a more conservative estimate than more common approaches, may overestimate uncertainty with so few included studies [46].

# Conclusions

The results of this systematic review and meta-analysis indicate that in a single session AVGs, when used for cardiovascular exercise, can evoke similar exercise intensities to those produced by traditional exercise modalities but are considered more enjoyable by subjects with respiratory diseases. However, little evidence exists from unsupervised long-term trials of AVGs for any rehabilitative purpose in respiratory conditions, though some limited evidence from supervised longer-term trials indicates that AVGs may be equal or slightly superior to traditional exercise training in some outcome measures.

Future research is needed regarding the long-term effects of AVGs, the use of AVGs for other rehabilitative purposes (such as strength training or airway clearance techniques), and the economic benefits of utilising AVGs rather than traditional supervised or unsupervised rehabilitation programs. In addition, future work could explore the differences between technologies (game consoles) and software (individual games) to enable the creation of more effective gaming interventions for use in rehabilitation. Finally, when assessing the enjoyment of game interventions validated outcome measures should be used to facilitate comparison to established research on enjoyment of exercise interventions.

# Acknowledgements

The authors thank Dr Michael David (University of Queensland, Brisbane, Australia) for his advice regarding the statistical methods used in this meta-analysis.

# References

1. Bateman ED, Hurd SS, Barnes PJ, Bousquet J, Drazen JM, FitzGeralde M, Gibson P, Ohta K, O’Byrne P, Pedersen SE, Pizzichini E, Sullivanee SD, Wenzel SE, Zar HJ. Global strategy for asthma management and prevention: GINA executive summary. Eur Respir J 2008;31(1):143–178. PMID:[18166595](http://www.ncbi.nlm.nih.gov/pubmed/18166595)

2. Rabe KF, Hurd S, Anzueto A, Barnes PJ, Buist SA, Calverley P, Fukuchi Y, Jenkins C, Rodriguez-Roisin R, Van Weel C, Zielinski J. Global strategy for the diagnosis, management, and prevention of chronic obstructive pulmonary disease: GOLD executive summary. Am J Respir Crit Care Med 2007;176(6):532–555. PMID:[17507545](http://www.ncbi.nlm.nih.gov/pubmed/17507545)

3. Cutting GR. Cystic fibrosis genetics: From molecular understanding to clinical application. Nat Rev Genet 2015;16(1):45–56. PMID:[25404111](http://www.ncbi.nlm.nih.gov/pubmed/25404111)

4. Maio S, Baldacci S, Carrozzi L, Pistelli F, Viegi G. The global burden of chronic respiratory diseases. Breathe [Internet] 2006;3(1):20–29. [doi: [10.1183/18106838.0301.20](https://doi.org/10.1183/18106838.0301.20)]

5. Carson KV, Chandratilleke MG, Picot J, Brinn MP, Esterman AJ, Smith BJ. Physical training for asthma. Cochrane Database Syst Rev 2013;CD001116. PMID:[24085631](http://www.ncbi.nlm.nih.gov/pubmed/24085631)

6. Radtke T, Nolan SJ, Hebestreit H, Kriemler S. Physical exercise training for cystic fibrosis. Cochrane Database Syst Rev 2015;6:CD002768. PMID:[26116828](http://www.ncbi.nlm.nih.gov/pubmed/26116828)

7. Gimeno-Santos E, Frei A, Steurer-Stey C, De Batlle J, Rabinovich RA, Raste Y, Hopkinson NS, Polkey MI, Van Remoortel H, Troosters T, Kulich K, Karlsson N, Puhan MA, Garcia- Aymerich J. Determinants and outcomes of physical activity in patients with COPD: A systematic review. Thorax 2014;69(8):731–739. PMID:[24558112](http://www.ncbi.nlm.nih.gov/pubmed/24558112)

8. McCarthy B, Casey D, Devane D, Murphy K, Murphy E, Lacasse Y. Pulmonary rehabilitation for chronic obstructive pulmonary disease. Cochrane Database Syst Rev 2015;CD003793. PMID:[25705944](http://www.ncbi.nlm.nih.gov/pubmed/25705944)

9. Holland AE, Wadell K, Spruit MA. How to adapt the pulmonary rehabilitation programme to patients with chronic respiratory disease other than COPD. Eur Respir Rev 2013;22(130):577–586. PMID:[24293474](http://www.ncbi.nlm.nih.gov/pubmed/24293474)

10. Rochester CL, Fairburn C, Crouch RH. Pulmonary rehabilitation for respiratory disorders other than chronic obstructive pulmonary disease. Clin Chest Med 2014 Jun;35(2):369–389. PMID:[24874132](http://www.ncbi.nlm.nih.gov/pubmed/24874132)

11. Button BM, Wilson C, Dentice R, Cox NS, Middleton A, Tannenbaum E, Bishop J, Cobb R, Burton K, Wood M, Moran F, Black R, Bowen S, Day R, Depiazzi J, Doiron K, Doumit M, Dwyer T, Elliot A, Fuller L, Hall K, Hutchins M, Kerr M, Lee AL, Mans C, O’Connor L, Steward R, Potter A, Rasekaba T, Scoones R, Tarrant B, Ward N, West S, White D, Wilson L, Wood J, Holland AE. Physiotherapy for cystic fibrosis in Australia and New Zealand: A clinical practice guideline. Respirology 2016;21(4):656–667. PMID:[27086904](http://www.ncbi.nlm.nih.gov/pubmed/27086904)

12. Qaseem A, Wilt TJ, Weinberger SE, Hanania NA, Criner G, Molen T van der, Marciniuk DD, Denberg T, Schünemann H, Wedzicha W, R. M, P. S. Diagnosis and management of stable chronic obstructive pulmonary disease: A clinical practice guideline update from the American College of Physicians, American College of Chest Physicians, American Thoracic Society, and European Respiratory Society. Ann Intern Med 2011;155(3):179–191. PMID:[21810710](http://www.ncbi.nlm.nih.gov/pubmed/21810710)

13. Caspersen CJ, Powell KE, Christenson GM. Physical activity, exercise, and physical fitness: Definitions and distinctions for health-related research. Public Health Rep 1985;100(2):126–131. PMID:[3920711](http://www.ncbi.nlm.nih.gov/pubmed/3920711)

14. Cox NS, Alison JA, Button BM, Wilson JW, Morton JM, Holland AE. Physical activity participation by adults with cystic fibrosis: An observational study. Respirology 2016;21(3):511–518. PMID:[26715596](http://www.ncbi.nlm.nih.gov/pubmed/26715596)

15. Hayton C, Clark A, Olive S, Browne P, Galey P, Knights E, Staunton L, Jones A, Coombes E, Wilson AM. Barriers to pulmonary rehabilitation: Characteristics that predict patient attendance and adherence. Resp Med 2013;107(3):401–407. PMID:[23261311](http://www.ncbi.nlm.nih.gov/pubmed/23261311)

16. Hogg L, Garrod R, Thornton H, McDonnell L, Bellas H, White P. Effectiveness, attendance, and completion of an integrated, system-wide pulmonary rehabilitation service for COPD: Prospective observational study. COPD 2012;9(5):546–554. PMID:[23030586](http://www.ncbi.nlm.nih.gov/pubmed/23030586)

17. Saunders T, Campbell N, Jason T, Dechman G, Hernandez P, Thompson K, Blanchard CM. Objectively measured steps/day in patients with chronic obstructive pulmonary disease: A systematic review and meta-analysis. J Phys Act Health 2016;13(11):1275–1283. PMID:[27334811](http://www.ncbi.nlm.nih.gov/pubmed/27334811)

18. Mancuso CA, Sayles W, Robbins L, Phillips EG, Ravenell K, Duffy C, Wenderoth S, Charlson ME. Barriers and facilitators to healthy physical activity in asthma patients. J Asthma 2006;43(2):137–143. PMID:[16517430](http://www.ncbi.nlm.nih.gov/pubmed/16517430)

19. O’Shea SD, Taylor NF, Paratz JD. … But watch out for the weather: Factors affecting adherence to progressive resistance exercise for persons with COPD. J Cardiopulm Rehabil Prev 2007;27(3):166–174. PMID:[17558200](http://www.ncbi.nlm.nih.gov/pubmed/17558200)

20. Swisher AK, Erickson M. Perceptions of physical activity in a group of adolescents with cystic fibrosis. Cardiopulm Phys Ther J 2008;19(4):107–114. PMID:[20467507](http://www.ncbi.nlm.nih.gov/pubmed/20467507)

21. Arnold E, Bruton A, Ellis-Hill C. Adherence to pulmonary rehabilitation: A qualitative study. Resp Med 2006;100(10):1716–1723. PMID:[16554147](http://www.ncbi.nlm.nih.gov/pubmed/16554147)

22. Hartman JE, ten Hacken NH, Boezen HM, de Greef MH. Self-efficacy for physical activity and insight into its benefits are modifiable factors associated with physical activity in people with COPD: A mixed-methods study. J Physiother 2013;59(2):117–124. PMID:[23663797](http://www.ncbi.nlm.nih.gov/pubmed/23663797)

23. Ferguson B, Baranowski T, Bingham P, Lieberman D, Medina E, Schell J, Yohannan SK. Health games come of age: An expert panel discussion. Games Health J 2012 Feb;1(1):11–17. PMID:[26196427](http://www.ncbi.nlm.nih.gov/pubmed/26196427)

24. Oh Y, Yang S. Defining exergames and exergaming. Proceedings of Meaningful Play 2010. pp. 1–17.

25. Pope Z, Zeng N, Gao Z. The effects of active video games on patients’ rehabilitative outcomes: A meta-analysis. Prev Med 2017;95:38–46. PMID:[27939260](http://www.ncbi.nlm.nih.gov/pubmed/27939260)

26. Staiano AE, Flynn R. Therapeutic uses of active videogames: A systematic review. Games Health J 2014;3(6):351–365. PMID:[26192642](http://www.ncbi.nlm.nih.gov/pubmed/26192642)

27. Bingham P, Lahiri T, Ashikaga T. Pilot trial of spirometer games for airway clearance practice in cystic fibrosis. Respir Care 2012;57(8):1278–1284. PMID:[22348602](http://www.ncbi.nlm.nih.gov/pubmed/22348602)

28. Oikonomou A, Hartescu D, Day D, Ma M. Computer games physiotherapy for children with cystic fibrosis. Virtual, Augmented Reality and Serious Games for Healthcare 1 Springer; 2014. pp. 411–443. [doi: [10.1007/978-3-642-54816-1_21](https://doi.org/10.1007/978-3-642-54816-1_21)]

29. Vilozni D, Barak A, Efrati O, Angarten A, Springer C, Yahav Y, Bentur L. The role of computer games in measuring spirometry in healthy and “asthmatic” preschool children. Chest 2005 Sep;128(3):1146–1155. PMID:[16162700](http://www.ncbi.nlm.nih.gov/pubmed/16162700)

30. Lange B, Flynn SM, Rizzo AA. Game-based telerehabilitation. Eur J Phys Rehabil Med 2009;45(1):143–151. PMID:[19282807](http://www.ncbi.nlm.nih.gov/pubmed/19282807)

31. Kuys SS, Hall K, Peasey M, Wood M, Cobb R, Bell SC. Gaming console exercise and cycle or treadmill exercise provide similar cardiovascular demand in adults with cystic fibrosis: A randomised cross-over trial. J Physiother 2011;57(1):35–40. PMID:[21402328](http://www.ncbi.nlm.nih.gov/pubmed/21402328)

32. Salonini E, Gambazza S, Meneghelli I, Tridello G, Sanguanini M, Cazzarolli C, Zanini A, Assael BM. Active video game playing in children and adolescents with cystic fibrosis: Exercise or just fun? Respir Care 2015;60(6):1172–1179. PMID:[25899477](http://www.ncbi.nlm.nih.gov/pubmed/25899477)

33. LeGear T, LeGear M, Preradovic D, Wilson G, Kirkham A, Camp PG. Does a Nintendo Wii exercise program provide similar exercise demands as a traditional pulmonary rehabilitation program in adults with COPD? Clin Respir J 2016;10(3):303–310. PMID:[25351282](http://www.ncbi.nlm.nih.gov/pubmed/25351282)

34. Rhodes RE, Fiala B, Conner M. A review and meta-analysis of affective judgments and physical activity in adult populations. Ann Behav Med 2009 Dec;38(3):180–204. PMID:[20082164](http://www.ncbi.nlm.nih.gov/pubmed/20082164)

35. Rhodes RE, Kates A. Can the affective response to exercise predict future motives and physical activity behavior? A systematic review of published evidence. Ann Behav Med 2015 Oct;49(5):715–731. PMID:[25921307](http://www.ncbi.nlm.nih.gov/pubmed/25921307)

36. Rhodes RE, Warburton DE, Bredin SS. Predicting the effect of interactive video bikes on exercise adherence: An efficacy trial. Psychol Health Med 2009 Dec;14(6):631–640. PMID:[20183536](http://www.ncbi.nlm.nih.gov/pubmed/20183536)

37. Lyons EJ, Tate DF, Ward DS, Ribisl KM, Bowling JM, Kalyanaraman S. Engagement, enjoyment, and energy expenditure during active video game play. Health Psychol 2014 Feb;33(2):174–81. PMID:[23527520](http://www.ncbi.nlm.nih.gov/pubmed/23527520)

38. Carbonera RP, Vendrusculo FM, Donadio MV. Physiological responses during exercise with video games in patients with cystic fibrosis: A systematic review. Resp Med 2016 Oct;119:63–69. [doi: [10.1016/j.rmed.2016.08.011](https://doi.org/10.1016/j.rmed.2016.08.011)]

39. Bonnechère B, Jansen B, Omelina L, Van Sint Jan S. The use of commercial video games in rehabilitation: A systematic review. Int J Rehabil Res 2016;39(4):277–290. PMID:[27508968](http://www.ncbi.nlm.nih.gov/pubmed/27508968)

40. Costa TH, Soares NM, Reis WA, Bublitz FM. A systematic review on the usage of games for healthcare. 2015 IEEE 5th International Conference on Consumer Electronics-Berlin (ICCE-Berlin) 2015. pp. 480–484. [doi: [10.1109/ICCE-Berlin.2015.7391316](https://doi.org/10.1109/ICCE-Berlin.2015.7391316)]

41. Ravenek KE, Wolfe DL, Hitzig SL. A scoping review of video gaming in rehabilitation. Disabil Rehabil Assist Technol 2016;11(6):445–453. PMID:[25815680](http://www.ncbi.nlm.nih.gov/pubmed/25815680)

42. Cochrane Airways. Search methods for the Cochrane Airways Group Specialised Register [Internet]. St George’s Hospital Medical School, London, UK; 2013. Available from: <http://airways.cochrane.org/sites/airways.cochrane.org/files/public/uploads/Search%20strategies%20document_2013_0.pdf>

43. Downs SH, Black N. The feasibility of creating a checklist for the assessment of the methodological quality both of randomised and non-randomised studies of health care interventions. J Epidemiol Community Health 1998;52(6):377–384. PMID:[9764259](http://www.ncbi.nlm.nih.gov/pubmed/9764259)

44. Hartung J, Knapp G. On tests of the overall treatment effect in meta-analysis with normally distributed responses. Stat Med 2001;20(12):1771–1782. PMID:[11406840](http://www.ncbi.nlm.nih.gov/pubmed/11406840)

45. Sidik K, Jonkman JN. A simple confidence interval for meta-analysis. Stat Med 2002;21(21):3153–3159. PMID:[12375296](http://www.ncbi.nlm.nih.gov/pubmed/12375296)

46. Cornell JE, Mulrow CD, Localio R, Stack CB, Meibohm AR, Guallar E, Goodman SN. Random-effects meta-analysis of inconsistent effects: A time for change. Ann Intern Med 2014;160(4):267–270. PMID:[24727843](http://www.ncbi.nlm.nih.gov/pubmed/24727843)

47. IntHout J, Ioannidis JP, Borm GF. The Hartung-Knapp-Sidik-Jonkman method for random effects meta-analysis is straightforward and considerably outperforms the standard DerSimonian-Laird method. BMC Med Res Methodol 2014;14(1):25. PMID:[24548571](http://www.ncbi.nlm.nih.gov/pubmed/24548571)

48. Hedges LV. Distribution theory for Glass’s estimator of effect size and related estimators. J Educ Behav Stat 1981;6(2):107–128. [doi: [10.3102/10769986006002107](https://doi.org/10.3102/10769986006002107)]

49. Walker E, Nowacki AS. Understanding equivalence and noninferiority testing. J Gen Intern Med 2011;26(2):192–196. PMID:[20857339](http://www.ncbi.nlm.nih.gov/pubmed/20857339)

50. Ries AL. Minimally clinically important difference for the UCSD Shortness of Breath Questionnaire, Borg scale, and visual analog scale. COPD 2005;2(1):105–110. PMID:[17136970](http://www.ncbi.nlm.nih.gov/pubmed/17136970)

51. Holland AE, Rasekaba T, Wilson JW, Button BM. Desaturation during the 3-minute step test predicts impaired 12-month outcomes in adult patients with cystic fibrosis. Respir Care 2011;56(8):1137–1142. PMID:[21496365](http://www.ncbi.nlm.nih.gov/pubmed/21496365)

52. Ruf K, Hebestreit H. Exercise-induced hypoxemia and cardiac arrhythmia in cystic fibrosis. J Cyst Fibros 2009;8(2):83–90. PMID:[19027369](http://www.ncbi.nlm.nih.gov/pubmed/19027369)

53. Casanova C, Cote C, Marin JM, Pinto-Plata V, de Torres JP, Aguirre-Jaíme A, Vassaux C, Celli BR. Distance and oxygen desaturation during the 6-min walk test as predictors of long-term mortality in patients with COPD. Chest 2008;134(4):746–752. PMID:[18625667](http://www.ncbi.nlm.nih.gov/pubmed/18625667)

54. Elbourne DR, Altman DG, Higgins JP, Curtin F, Worthington HV, Vail A. Meta-analyses involving cross-over trials: Methodological issues. Int J Epidemiol 2002;31(1):140–149. PMID:[11914310](http://www.ncbi.nlm.nih.gov/pubmed/11914310)

55. Wan X, Wang W, Liu J, Tong T. Estimating the sample mean and standard deviation from the sample size, median, range and/or interquartile range. BMC Med Res Methodol 2014;14(1):135. PMID:[25524443](http://www.ncbi.nlm.nih.gov/pubmed/25524443)

56. Viechtbauer W. Conducting meta-analyses in R with the metafor package. J Stat Softw 2010;36(3):1–48. [doi: [10.18637/jss.v036.i03](https://doi.org/10.18637/jss.v036.i03)]

57. Baumer B, Cetinkaya-Rundel M, Bray A, Loi L, Horton NJ. R markdown: Integrating a reproducible analysis tool into introductory statistics. arXiv preprint arXiv:14021894 2014;

58. Moher D, Liberati A, Tetzlaff J, Altman DG, The PRISMA Group. Preferred reporting items for systematic reviews and meta-analyses: The PRISMA statement. PLoS Med 2009 Jul;6(7):e1000097. PMID:[19621072](http://www.ncbi.nlm.nih.gov/pubmed/19621072)

59. del Corral T, Percegona J, Seborga M, Rabinovich R, Vilaró J. Physiological response during activity programs using Wii-based video games in patients with cystic fibrosis (CF). J Cyst Fibros 2014;13(6):706–711. PMID:[24935613](http://www.ncbi.nlm.nih.gov/pubmed/24935613)

60. del Corral T, Cebrià i Iranzo MÀ, López-de-Uralde-Villanueva I, Martínez-Alejos R, Blanco I, Vilaró J. Effectiveness of a home-based active video game programme in young cystic fibrosis patients. Respiration 2017; PMID:[29045949](http://www.ncbi.nlm.nih.gov/pubmed/29045949)

61. Holmes H, Wood J, Jenkins S, Winship P, Lunt D, Bostock S, Hill K. Xbox KinectTM represents high intensity exercise for adults with cystic fibrosis. J Cyst Fibros 2013;12(6):604–608. PMID:[23746575](http://www.ncbi.nlm.nih.gov/pubmed/23746575)

62. Albores J, Marolda C, Haggerty M, Gerstenhaber B, ZuWallack R. The use of a home exercise program based on a computer system in patients with chronic obstructive pulmonary disease. J Cardiopulm Rehabil Prev 2013;33(1):47–52. [doi: [10.1097/HCR.0b013e3182724091](https://doi.org/10.1097/HCR.0b013e3182724091)]

63. Wardini R, Dajczman E, Yang N, Baltzan M, Prefontaine D, Stathatos M, Marciano H, Watson S, Wolkove N. Using a virtual game system to innovate pulmonary rehabilitation: Safety, adherence and enjoyment in severe chronic obstructive pulmonary disease. Can Respir J 2013;20(5):357–361. [doi: [10.1155/2013/563861](https://doi.org/10.1155/2013/563861)]

64. Gomes E, Carvalho CRF, Peixoto-Souza FS, Teixeira-Carvalho EF, Mendonca JFB, Stirbulov R, Sampaio LMM, Costa D. Active video game exercise training improves the clinical control of asthma in children: Randomized controlled trial. PLoS ONE 2015 Aug;10(8):e0135433. PMID:[26301706](http://www.ncbi.nlm.nih.gov/pubmed/26301706)

65. Hoffman AJ, Brintnall RA, Brown JK, von Eye A, Jones LW, Alderink G, Ritz-Holland D, Enter M, Patzelt LH, VanOtteren GM. Too sick not to exercise: Using a 6-week, home-based exercise intervention for cancer-related fatigue self-management for postsurgical non-small cell lung cancer patients. Cancer Nurs 2013;36(3):175–188. PMID:[23051872](http://www.ncbi.nlm.nih.gov/pubmed/23051872)

66. Mazzoleni S, Montagnani G, Vagheggini G, Buono L, Moretti F, Dario P, Ambrosino N. Interactive videogame as rehabilitation tool of patients with chronic respiratory diseases: Preliminary results of a feasibility study. Resp Med 2014 Oct;108(10):1516–24. PMID:[25087837](http://www.ncbi.nlm.nih.gov/pubmed/25087837)

67. Cohen J. Statistical Power Analysis for the Behavioral Sciences. Second Edition. Hillsdale, USA: Lawrence Erlbaum Associated, Inc. 1988. ISBN:978-0805802832

68. Garber CE, Blissmer B, Deschenes MR, Franklin BA, Lamonte MJ, Lee I-M, Nieman DC, Swain DP. Quantity and quality of exercise for developing and maintaining cardiorespiratory, musculoskeletal, and neuromotor fitness in apparently healthy adults: Guidance for prescribing exercise. Med Sci Sports Exerc 2011;43(7):1334–1359. PMID:[21694556](http://www.ncbi.nlm.nih.gov/pubmed/21694556)

69. Hoffman AJ, Brintnall RA, Brown JK, von Eye A, Jones LW, Alderink G, Ritz-Holland D, Enter M, Patzelt LH, VanOtteren GM. Virtual reality bringing a new reality to postthoracotomy lung cancer patients via a home-based exercise intervention targeting fatigue while undergoing adjuvant treatment. Cancer Nurs 2014;37(1):23–33. PMID:[23348662](http://www.ncbi.nlm.nih.gov/pubmed/23348662)

70. O’Donovan C, Greally P, Canny G, McNally P, Hussey J. Active video games as an exercise tool for children with cystic fibrosis. J Cyst Fibros 2014;13(3):341–346. PMID:[24189057](http://www.ncbi.nlm.nih.gov/pubmed/24189057)

71. Peng W, Lin J-H, Crouse J. Is playing exergames really exercising? A meta-analysis of energy expenditure in active video games. Cyberpsychol Behav Soc Netw 2011;14(11):681–688. PMID:[21668370](http://www.ncbi.nlm.nih.gov/pubmed/21668370)

72. Gao Z, Chen S, Pasco D, Pope Z. A meta-analysis of active video games on health outcomes among children and adolescents. Obes Rev 2015;16(9):783–794. PMID:[25943852](http://www.ncbi.nlm.nih.gov/pubmed/25943852)

73. Segal KR, Dietz WH. Physiologic responses to playing a video game. Am J Dis Child 1991;145(9):1034–1036. PMID:[1877563](http://www.ncbi.nlm.nih.gov/pubmed/1877563)

74. Wang X, Perry AC. Metabolic and physiologic responses to video game play in 7-to 10-year-old boys. Arch Pediatr Adolesc Med 2006;160(4):411–415. PMID:[16585487](http://www.ncbi.nlm.nih.gov/pubmed/16585487)

75. Sherman JD, Sherman MS, Heiman-Patterson T. Cardiopulmonary response to videogaming: Slaying monsters using motion sensor versus joystick devices. Games Health J 2014;3(5):284–290. [doi: [10.1089/g4h.2014.0049](https://doi.org/10.1089/g4h.2014.0049)]

76. Capodaglio E. Comparison between the CR10 Borg’s scale and the VAS (visual analogue scale) during an arm-cranking exercise. J Occup Rehabil 2001;11(2):69–74. PMID:[11706532](http://www.ncbi.nlm.nih.gov/pubmed/11706532)

77. Wilson RC, Jones P. A comparison of the visual analogue scale and modified Borg scale for the measurement of dyspnoea during exercise. Clin Sci 1989;76(3):277–282. PMID:[2924519](http://www.ncbi.nlm.nih.gov/pubmed/2924519)

78. Jenkins S, Čečins N. Six-minute walk test: Observed adverse events and oxygen desaturation in a large cohort of patients with chronic lung disease. Intern Med J 2011;41(5):416–422. PMID:[20059599](http://www.ncbi.nlm.nih.gov/pubmed/20059599)

79. Kendzierski D, DeCarlo KJ. Physical activity enjoyment scale: Two validation studies. J Sport Exerc Psychol 1991;13(1):50–64. [doi: [10.1123/jsep.13.1.50](https://doi.org/10.1123/jsep.13.1.50)]

80. Hardy CJ, Rejeski WJ. Not what, but how one feels: The measurement of affect during exercise. J Sport Exerc Psychol 1989;11(3):304–317. [doi: [10.1123/jsep.11.3.304](https://doi.org/10.1123/jsep.11.3.304)]

81. Williams DM, Dunsiger S, Jennings EG, Marcus BH. Does affective valence during and immediately following a 10-min walk predict concurrent and future physical activity? Ann Behav Med 2012;44(1):43–51. PMID:[22532005](http://www.ncbi.nlm.nih.gov/pubmed/22532005)

82. Thum JS, Parsons G, Whittle T, Astorino TA. High-intensity interval training elicits higher enjoyment than moderate intensity continuous exercise. PloS ONE 2017;12(1):1–11. PMID:[28076352](http://www.ncbi.nlm.nih.gov/pubmed/28076352)

83. Bartlett JD, Close GL, MacLaren DP, Gregson W, Drust B, Morton JP. High-intensity interval running is perceived to be more enjoyable than moderate-intensity continuous exercise: Implications for exercise adherence. J Sports Sci 2011;29(6):547–553. PMID:[21360405](http://www.ncbi.nlm.nih.gov/pubmed/21360405)
