# Supplementary material for: Active Video Games for Rehabilitation in Respiratory Conditions: Systematic Review and Meta-Analysis
Source: JMIR Serious Games. 2019 Feb 25;7(1):e10116. doi: 10.2196/10116 (PMC6409512; doi:10.2196/10116)
Supplement: Multimedia Appendix 5 [file games_v7i1e10116_app5.pdf]

**Table 2:** Characteristics of included studies

| Author (Year) | Population | Participants | Intervention | Duration | Outcome Measures | Results |
|---|---|---|---|---|---|---|
| Albores (2013) | COPD | n=25 (Exp = 25)<br>Age (yr) = 68±10 | Exp = Nintendo Wii™ (*Wii Fit*™) | 12 week unsupervised home exercise: >30 minutes per day, most days of the week | a) ESWT<br>b) Arm lifts<br>c) STS<br>d) Dyspnoea (MRC, CRQ)<br>e) Total play time | ESWT improved (*P*=.005) by 131±183s after 12 weeks of training, Significant increases in sit-to stands (*P*=.01) and arm lifts (*P*=.02) in 60s. |
| Bingham (2012) | CF | n=19 (Exp/Con = 19)<br>Age (yr) = 9.3±1.3 | Exp = Custom spirometer games<br>Con = Non-game spirometer program | 2-3 weeks unsupervised home exercise. Ad libitum exercise with daily prompting. | a) FEV1<br>b) VC<br>c) Days used (of days available)<br>d) Minutes used (per day available) | FEV1, as a % of predicted FEV1, was better maintained (*P*=.01) in Exp, per day used, than in Con (0.3±2.4 vs -2.5±5.2%/day). There was no significant difference in minutes used per day with the Exp vs Con (*P*=.07). |
| del Corral (2014) | CF | n=24 (Exp/Con = 24)<br>Age (yr) = 12.6±3.7 | Exp1 =Nintendo Wii™ (*Wii Fit*™ *Plus*)<br>Exp2= Nintendo Wii™ (*EA Sports Active*™)<br>Exp3= Nintendo Wii™ (*Family Trainer Extreme Challenge*)<br>Con= 6MWT | Single session:<br>Exp1-3 = 5 minutes<br>Con = 6 minutes | a) HR (max)<br>b) Dyspnoea (11-point Borg)<br>c) $SpO_2$<br>d) $VO_2$<br>e) VE | $VO_2$ was higher (*P*<.0001) in Exp2 (1232.2±427.2ml/min) and Exp3 (1252.6±360.2 ml/min) than in Con (1024.2±282.2ml/min) during the last 3 minutes. $VO_2$ and VE were lower in Exp1 than Con. No difference in dyspnoea or $SpO_2$ between interventions. |
| del Corral (2017) | CF | n=40 (Exp = 20, Con = 20)<br>Age (yr) = Exp 12.6±3.1; Con 11±3 | Exp = Nintendo Wii™ (*EA Sports Active*™ 2)<br>Con = usual care (no intervention) | Exp = 6 weeks unsupervised home exercise; 30-60 minute sessions, 5 day per week | a) MSWT<br>b) 6MWT<br>c) Horizontal jump test<br>d) Medicine ball throw<br>e) Grip strength<br>f) CFQ-R<br>g) Adherence (Exp only) | In Exp, MSWT distance improved by 58.95±55.22m (*P*<.05) and 6MWT distance improved by 30.95±15.44m (*P*<.01). Con showed no improvement. Horizontal jump distance, medicine ball throw distance, and |

| | | | | | | |
|---|---|---|---|---|---|---|
| | | | | 12 month follow-up | | grip strength improved in Exp ($p < 0.01$) but not in Con. CFQ physical activity subsection improved in Exp ($P<.05$) but not in Con. Adherence to Exp was 95% in the 6-week intervention, and 35% in the 12-month followup. Except for 6MWT distance and horizontal jump distance, all improvements were maintained over 12-month followup. |
| Gomes (2015) | Asthma | n=36 (Exp = 20, Con = 16)<br>Age (yr) = Exp 7.5±1.9; Con 8.0±2.0 | Exp = Microsoft Xbox 360 Kinect™ (*Kinect™ Adventures! - Reflex Ridge*)<br>Con = treadmill | 8 weeks supervised exercise: 40 minute sessions, 2 days per week | a) MET<br>b) Energy expenditure<br>c) VO2 max<br>d) HR (average)<br>e) FEV1/FVC<br>f) FeNO | Total energy expenditure was higher ($P<.01$) in Exp (159.9±41.6cal) vs Con (133.3±32.1cal). Both Exp and Con showed a significant improvement in $VO_2$ max after training. FeNO was significantly reduced in Exp ($P=.04$) but not in Con ($P=.64$). |
| Hoffman (2013), Hoffman (2014) | Lung Cancer (Postthoracotomy) | n=7 (Exp =7)<br>Age (yr) = 64.6±6.5 | Exp = Nintendo Wii™ (*Wii Fit™ Plus*) | 6 weeks of unsupervised home exercise: 5 minutes per day in week 1 to 30 minutes per day in week 7; 10 week follow-up assessment | a) Steps/day | Steps per day changed from 4650±3105 in week 1 to 6393±3752 in week 6. |
| Holmes (2013) | CF | n=10 (Exp/Con = 10)<br>Age (yr) = 29±6 | Exp = Microsoft Xbox Kinect™ (*Your Shape™: Fitness Evolved*)<br>Con= cycle ergometer | Single session:<br>Exp = 20 minutes<br>Con = 8-12 minutes | a) %max HR achieved<br>b) % of HR achieved in control<br>c) $SpO_2$<br>d) Dyspnoea (11-point Borg) | Mean HR in the final 10 minutes of Exp was 86% (95% CI [81,92]) of the peak HR in Con. |

| | | | | | | |
|---|---|---|---|---|---|---|
| Kuys (2011) | CF | n=19 (Exp/Con = 19)<br>Age (yr) = 28±7 | Exp = Nintendo Wii™ (*EA Sports Active*™)<br>Con= treadmill or stationary cycle | Single session:<br>Exp/Con = 15 minutes | a) HR (average, max, min)<br>b) MET<br>c) Energy expenditure<br>d) $SpO_2$<br>e) Enjoyment<br>f) RPE<br>g) Dyspnoea (11-point Borg)<br>h) Perceived feasibility | No significant differences in interventions in mean HR (MD=3bpm, 95% CI [-3, 9] or METs (MD=0.1, 95% CI [-0.3,0.5]. Exp was significantly more enjoyable than Con (MD=2.6cm, 95% CI [1.6, 3.6]. |
| LeGear (2016) | COPD | n=10 (Exp/Con = 10)<br>Age (yr) = 65±9 | Exp = Nintendo Wii™ (*EA Sports Active*™)<br>Con= treadmill | Single session:<br>Exp/Con = 15 minutes | a) HR (average)<br>b) EE<br>c) $SpO_2$<br>d) RPE<br>e) Dyspnoea (11-point Borg)<br>f) Enjoyment | No significant differences in interventions in mean HR (MD=-0.167bpm, 95% CI [--4.83, 4.50]) or EE (MD=36.3J, 95% CI [-31.4, 104]) between Exp and Con. 50% strongly agreed Exp was enjoyable, compared to 11% for Con. |
| Mazzoleni (2014) | Chronic respiratory diseases (mostly COPD) | n=40 (Exp = 20, Con = 20)<br>Age (yr) = Exp 68.9±11; Con 73.5±9.2 | Exp = usual pulmonary rehabilitation + Nintendo Wii™ (*Wii Fit*™ *Plus*)<br>Con = usual pulmonary rehabilitation alone | 3 weeks supervised exercise: Exp = 7 daily 30 minute game sessions in final week | a) 6MWT<br>b) Arm cycle power<br>c) Leg cycle power<br>d) MIP/MEP<br>e) Program acceptability | 6MWT improved more (*P*=.028) in Exp (97.4±64.8m) vs Con (61.1±28.3m). Dyspnoea also showed more improvement in Exp than the Con (*P*<.001). |
| Salonini (2015) | CF | n=30 (Exp/Con = 30)<br>Age (yr) = 12±5 | Exp = Microsoft Xbox 360 Kinect™ (*Kinect*™ *Adventures! - River Rush*)<br>Con = stationary cycle | Single session:<br>Exp/Con = 20 minutes | a) HR (max)<br>b) % HR target reached<br>c) $SpO_2$<br>d) Dyspnoea (modified VAS)<br>e) Enjoyment | No significant difference (*P*=.2) in the maximum HR reached in Exp (165.0±23.6bpm) vs Con (170.8±13.2bpm). Exp was rated lower in dyspnoea (*P*<.001) and higher in enjoyment (*P*<.001) than Con. |
| Wardini (2013) | COPD | n=32 (Exp = 32)<br>Age (yr) = 66±9 | Exp = Nintendo Wii™ (*Wii Sports*™, *Wii Sports Resort*™, *Wii Fit*™ *Plus*) | 3-4 weeks supervised exercise: Offered 3/week, average | a) HR (average)<br>b) Dyspnoea (modified Borg)<br>c) $SpO_2$<br>d) Adherence<br>e) Attendance<br>f) Enjoyment | HR and dyspnoea increased from 88.1±14.8bpm and 1.5±1.1 (modified Borg scale) at rest to 101.9±18bpm and 3.2±1.2 |

| | | | |
|---|---|---|---|
| | | session length = 14 minutes | during the game session compared to *P*<.001 for both), while $SpO_2$ dropped from 93.7±2.8% to 90.7±4.6% (*P*<.001). Adherence and attendance was 76% and 64±35%, compared to 100% and 88±13% for conventional PR. |

COPD = chronic obstructive pulmonary disease, CF = cystic fibrosis, n = sample size, Exp = experimental group (active video game), Con = control group, 6MWT = 6-minute walk test, ISWT = incremental shuttle walk test, ESWT = endurance shuttle walk test, STS = sit-to-stand, MRC = Medical Research Council dyspnoea rating, CRQ = chronic respiratory questionnaire, FEV1 = forced expiratory volume in 1 second, VC = vital capacity, HR = heart rate, $SpO_2$ = peripheral blood oxygen saturation, $VO_2$ = volume of oxygen consumption, VE = exhaled minute volume, BPM = beats per minute, MSWT = modified shuttle walk test, CFQ-R = Cystic Fibrosis Questionnaire Revised, MET = metabolic equivalent, FVC = forced vital capacity, FeNO = fraction of exhaled nitric oxide, RPE = rating of perceived exertion, EE = energy expenditure, MIP = maximal inspiratory pressure, MEP = maximal expiratory pressure, CI = confidence interval, VAS = visual analogue scale
