# Supplementary material for: Active Video Games for Rehabilitation in Respiratory Conditions: Systematic Review and Meta-Analysis
Source: JMIR Serious Games. 2019 Feb 25;7(1):e10116. doi: 10.2196/10116 (PMC6409512; doi:10.2196/10116)
Supplement: Multimedia Appendix 7 [file games_v7i1e10116_app7.pdf]

Appendix 7: Sensitivity analysis

A sensitivity analysis, conducted by altering the assumed correlation coefficients of cross-over studies, indicated that this assumption had negligible influence on the outcomes measures of heart rate and enjoyment, and little influence on the results of dyspnoea.

Outcome variable	Correlation	Pooled Estimate, HKSJ method (95% CI)
Heart rate	0.0	1.718 [-13.837, 17.272]
	0.613(from Kuys 2011)	1.436 [-14.311, 17.184]
	1.0	1.257 [-14.603, 17.117]
Dyspnoea	0.0	0.283 [-0.176, 0.743]
	0.833(from Kuys 2011)	0.433 [-0.793, 1.66]
	1.0	0.476 [-0.797, 1.749]
SpO_2	0.0	1.162 [-1.86, 4.183]
	0.761(from Kuys 2011)	1.121 [-1.915, 4.157]
	1.0	1.108 [-1.931, 4.148]
